# Evolution of Phototransduction Genes in Lepidoptera

**DOI:** 10.1093/gbe/evz150

**Published:** 2019-07-12

**Authors:** Aide Macias-Muñoz, Aline G Rangel Olguin, Adriana D Briscoe

**Affiliations:** Department of Ecology and Evolutionary Biology, University of California, Irvine

**Keywords:** opsin, trp, DAGL, wunen, Calx, Nckx30C

## Abstract

Vision is underpinned by phototransduction, a signaling cascade that converts light energy into an electrical signal. Among insects, phototransduction is best understood in *Drosophila melanogaster.* Comparison of *D. melanogaster* against three insect species found several phototransduction gene gains and losses, however, lepidopterans were not examined. Diurnal butterflies and nocturnal moths occupy different light environments and have distinct eye morphologies, which might impact the expression of their phototransduction genes. Here we investigated: 1) how phototransduction genes vary in gene gain or loss between *D. melanogaster* and Lepidoptera, and 2) variations in phototransduction genes between moths and butterflies. To test our prediction of phototransduction differences due to distinct visual ecologies, we used insect reference genomes, phylogenetics, and moth and butterfly head RNA-Seq and transcriptome data. As expected, most phototransduction genes were conserved between *D. melanogaster* and Lepidoptera, with some exceptions. Notably, we found two lepidopteran opsins lacking a *D. melanogaster* ortholog. Using antibodies we found that one of these opsins, a candidate retinochrome, which we refer to as unclassified opsin (UnRh), is expressed in the crystalline cone cells and the pigment cells of the butterfly, *Heliconius melpomene*. Our results also show that butterflies express similar amounts of *trp* and *trpl* channel mRNAs, whereas moths express ∼50× less *trp*, a potential adaptation to darkness. Our findings suggest that while many single-copy *D. melanogaster* phototransduction genes are conserved in lepidopterans, phototransduction gene expression differences exist between moths and butterflies that may be linked to their visual light environment.

## Introduction

Vision has intrigued scientists for many years. One of the earliest steps in vision involves the conversion of light into an electrical signal, a process known as phototransduction (Shichida and Matsuyama 2009). Phototransduction is one of the best-studied signaling pathways. In *Drosophila melanogaster*, phototransduction genes have been investigated for over 40 years (Hardie 2001; Hardie and Raghu 2001; Katz and Minke 2009; Montell 2012; Hardie and Juusola 2015). However, studies of phototransduction genes in other insects are largely lacking. A comparison of vision-related genes in four insect genomes (mosquito, red flour beetle, honeybee, and fruit fly) found gains and losses across lineages (Bao and Friedrich 2009). *Drosophila melanogaster* had by far the largest number of gene gains compared with the other insects. This implies that those insects missing *D. melanogaster* orthologs may differ in the genes underlying phototransduction. 

**Fig. 1.**
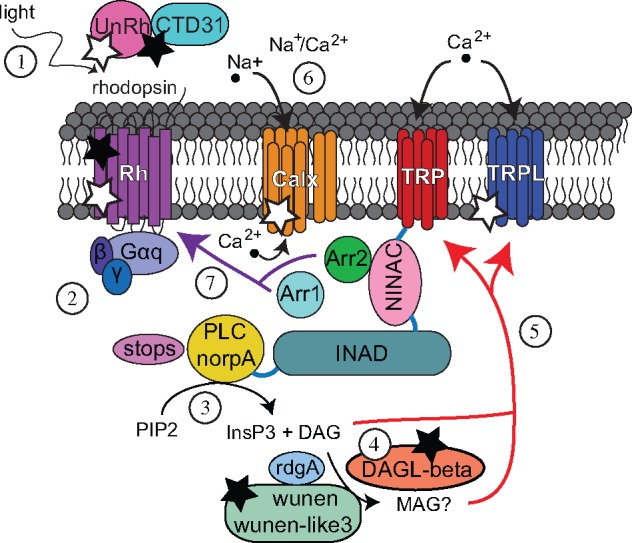
—Speculative model of the lepidopteran phototransduction cascade. Most lepidopteran vision genes are single-copy orthologs of *D. melanogaster* genes except in chromophore binding, photoisomerization, and diacylglycerol metabolism. 1) Light activates rhodopsin by a conformational change of the chromophore from 11-*cis* 3-hydroxyretinal to all-*trans*. The chromophore is transported by Hme CTD31 and photoisomerized from all-*trans* 3-hydroxyretinal to 11-*cis* by the unclassified opsin (UnRh), orthologs of which are not found in *D. melanogaster*. 2) G*α*q is released from a G-protein complex of three subunits (*α*, *β*, and *γ*) and activates phospholipase C (PLC). 3) PLC hydrolyzes PIP_2_ to produce inositol 1,4,5-trisphosphate (InsP_3_) and diacylglycerol (DAG). 4) Diacylglycerol lipase (DAGL*β*) hydrolyzes DAG to produce MAG. In *D. melanogaster*, DAGL*α* (inaE) hydrolyzes DAG. DAG levels are also regulated by rdgA and, perhaps, wunen or wunen-like 3. In *D. melanogaser*, lazaro plays the latter role. 5) DAG and MAG may activate TRP and TRPL by a mechanism that has not been established. A signaling complex that includes TRP and PLC is coordinated by INAD. 6) Na^+^/Ca^2+^ exchanger channel (Calx) pumps Ca^2+^ out of the photoreceptor cell. 7) Arrestin 1 and 2 bind rhodopsin to terminate the cascade with Arrestin 2 as the dominant arrestin in both *D. melanogaster* and butterflies. INAD and NinaC bind to each other, and both bind calmodulin, to accelerate arrestin binding rhodopsin. STOPS is another protein that terminates phototransduction. Black stars signify differences in phototransduction between *D. melanogaster* and Lepidoptera due to gene duplication. White stars represent differences in relative expression of *UnRh*, *Rh7*, and *Calx* between moths and butterflies, and in *trp* and *trpl* between flies, moths, and butterflies. Further description of these genes can be found in supplementary Table S11, Supplementary Material online.

Phototransduction takes place in specialized neurons known as photoreceptor cells whose microvilli incorporate light-sensitive opsin proteins bound to a retinal-derived molecule called a chromophore (Fain et al. 2010). Phototransduction begins when light is absorbed by the chromophore (11-*cis*-3-hydroxyretinal in *D. melanogaster*) causing the chromophore to change its conformation from *cis-* to all-*trans* (von Lintig et al. 2010). In *D. melanogaster*, this change in conformation triggers a G-protein-coupled cascade (similar to fig. 1) that activates phospholipase C (PLC) (Bloomquist et al. 1988). PLC hydrolyzes phosphatidylinositol 4,5-bisphosphate (PIP_2_) to produce inositol 1,4,5-trisphosphate (InsP_3_) and diacylglycerol (DAG) (Bloomquist et al. 1988; Hardie 2001). Concurrently, by a mechanism that is not well understood, there is an opening of Ca^2+^-permeable light-sensitive transient receptor potential (TRP) and transient receptor potential-like (TRPL) channels, which causes depolarization of the cell (Montell and Rubin 1989; Hardie and Minke 1992; Niemeyer et al. 1996; Shieh and Zhu 1996; Montell 2005). Finally, phototransduction is terminated when the activated rhodopsin (metarhodopsin) binds arrestin (Dolph et al. 1993; Stavenga and Hardie 2011).

A plethora of studies have focused on characterizing the opsins including their expression in photoreceptor cells and the arrangement of those photoreceptor cells across the compound eye (Spaethe and Briscoe 2005; Henze et al. 2012; Futahashi et al. 2015; McCulloch et al. 2016; Perry et al. 2016; Giraldo-Calderón et al. 2017; McCulloch et al. 2017). Opsin phylogenies have been used to understand the evolutionary history of light detection (Arendt 2003; Raible et al. 2006; Plachetzki et al. 2007; Suga et al. 2008; Porter et al. 2012; Ramirez et al. 2016; Vöcking et al. 2017). These studies have reconstructed opsins present in the ancestor of bilaterian animals (Ramirez et al. 2016) and have described new opsin types (Vöcking et al. 2017). However, despite the large focus on opsins, changes in the downstream pathway in which opsins function undoubtedly contribute to differences in vision (Plachetzki et al. 2010). Fewer studies have investigated the downstream phototransduction cascade in non-*D. melanogaster* insects. Studies of phototransduction in other insects have focused on presence, absence, or relative expression of genes in head transcriptomes. In the troglobiont beetle, *Ptomaphagus hirtus*, for example, 20 genes were identified from adult head mRNA (Friedrich et al. 2011). Exposure of the oriental armyworm, *Mythimna separata*, to different light environments resulted in differential expression of phototransduction genes in adult heads (Duan et al. 2017). Similarly, phototransduction genes were also differentially expressed (DE) between seasonal forms in heads of the butterfly *Bicyclus anynana* (Macias-Muñoz et al. 2016). One study quantified opsin and TRP channel gene expression and used RNAi to determine that *trpl* has the largest effect on phototransduction in the nocturnal cockroach *Periplaneta americana* (French et al. 2015). Yet, it remains largely unknown how variable the phototransduction cascade is between insect species. 

Lepidoptera provides an interesting group in which to investigate the molecular evolution and expression of phototransduction genes in insects adapted to different light environments (Yagi and Koyama 1963; Horridge et al. 1972; Nilsson et al. 1984; Yack et al. 2007; Warrant and Dacke 2016). Unlike *D. melanogaster*, in which an ommatidium consists of eight photoreceptors with an open rhabdom, the microvillar stacks where light is absorbed by the rhodopsins (Wernet et al. 2015), butterflies have nine photoreceptor cells and a fused rhabdom (Wernet et al. 2015). Interestingly, moths and butterflies also differ from each other in eye morphology related to their light environments. Most butterflies have apposition-type eyes, where light from each lens is processed by one rhabdom and each ommatidium is separated by a sheath of light-absorbing screening pigment which blocks stray light from other ommatidia (Yack et al. 2007; Warrant and Dacke 2016; Conversely, moths have superposition-type eyes where rhabdoms are separated from the crystalline cones by a translucent area allowing light to reach each rhabdom from hundreds of lenses (Yack et al. 2007; Warrant and Dacke 2016).

We predicted that we would find variation in phototransduction gene gains and losses between *D. melanogaster* and Lepidoptera, and between moths and butterflies due to differences in eye morphology. In fact, phylogenetic analyses have revealed numerous duplications of lepidopteran opsin genes (Spaethe and Briscoe 2004; Sison-Mangus et al. 2008; Briscoe et al. 2010). A survey of 23 vision-related gene families in 19 metazoan genomes revealed that eye development and phototransduction genes have higher rates of retention and duplications in pancrustaceans (Rivera et al. 2010). Because only the nocturnal domesticated silkmoth *Bombyx mori* was used in the pancrustacean study and only five gene families involved in phototransduction were examined (r-opsin, TRP, PLC, Gq-alpha, and arrestin) (Rivera et al. 2010), it remains to be seen if there are additional differences in phototransduction genes between *D. melanogaster* and moth and butterfly species. In our present study, we expand the genes surveyed thus far by looking at 76 phototransduction-related genes. Phylogenetic analyses of phototransduction genes in Lepidoptera may reveal: 1) the extent to which *D. melanogaster* phototransduction genes are duplicated or deleted in Lepidoptera, 2) lepidopteran-specific phototransduction features, and 3) differences between moths and butterflies.

Although gene trees tell the probable evolutionary history of gene families, gene expression data provide a step toward inferring gene function. Genes involved in vision should be highly expressed in photoreceptor cells and upregulated in the eyes relative to other tissue types. Visualizing or quantifying where phototransduction genes are expressed will reveal whether they have a potential role in vision. As an example, the horseshoe crab *Limulus polyphemus* has 18 opsins, some of which are expressed only in the eyes, in eyes and central nervous system, exclusively in the central nervous system, and some not expressed in either (Battelle et al. 2016). It is possible that the opsins missing from the eyes and central nervous system are expressed in other tissue types and have nonvisual functions (Feuda et al. 2016) or are not expressed at all. Similarly, the reference genome of the butterfly *Heliconius melpomene* (Davey et al. 2016) has a *UVRh* duplication but mRNA levels of one copy are downregulated in adult eyes compared with the other copy, and no protein expression of the downregulated copy is detectable in the eye (McCulloch et al. 2017). Studies such as these highlight the importance of quantifying gene expression in candidate tissues before inferring gene function based on sequence alone. Furthermore, it is also possible that a paralog has assumed the predicted visual function. As an example, *H. melpomene* is missing an ortholog of *D. melanogaster* chromophore-binding *pinta* (Smith and Briscoe 2015; Wang and Montell 2005). Instead, a lepidopteran paralog (*CTD31*) appears to carry out a similar function to that of the missing gene (Macias-Muñoz et al. 2017). Moreover, as observed in the cockroach, whereas genes such as *trp* and *trpl* are conserved and expressed, one gene copy (*trpl*) might have a greater impact on phototransduction than the other (French et al. 2015). Consequently, investigating both gene gain/loss and the expression of phototransduction genes in Lepidoptera might uncover differences in visual processing that helps moths and butterflies function in different light environments.

In this study, we combined transcriptomics and phylogenetics to perform an extensive investigation of candidate phototransduction genes in Lepidoptera. We used RNA-Sequencing data from four tissues of the butterfly *H. melpomene* to identify genes upregulated in heads. We hypothesized that genes upregulated in heads might have eye and vision-related functions. A functional enrichment analysis suggested that many of the genes upregulated in *H. melpomene* heads function in phototransduction. To identify gene gain or loss between *D. melanogaster* and Lepidoptera, and between moths and butterflies, we extracted 76 phototransduction-related gene sequences from reference genomes of eight insect species including the moth, *Manduca sexta*, and the butterflies, *Danaus plexippus* and *H. melpomene* (Zhan et al. 2011; Davey et al. 2016; Kanost et al. 2016). Then we generated 32 phylogenetic trees. In case any genes were missing annotations in the reference assemblies, we searched *de novo* transcriptome assemblies from *M. sexta*, *H. melpomene*, and *D. plexippus*. We found that most of the phototransduction pathway is conserved between Lepidoptera and *D. melanogaster*, with some exceptions (see stars in fig. 1). Our methods allowed us to uncover two lepidopteran opsin genes that lack a homolog in *D. melanogaster*. One of the opsins was highly expressed in butterfly eyes so we used antibodies to locate its expression in pigment cells. In addition, DAG regulation appears to differ between Lepidoptera and *D. melanogaster*, where a paralogous gene in lepidopterans, *DAGβ*, may be taking on a role of a lost ortholog of *D. melanogaster, DAGα*. Although we found a few gene duplication differences between moth and butterfly species, we did not find any consistent differences in gene duplications between the moths and butterflies investigated. Instead, we discovered an intriguing difference between moths and butterflies in their expression of vision-related ion channels, *trp*, *Calx*, and *Nckx30C*.

## Materials and Methods

### Transcriptome-Wide Differential Expression Analysis

RNA-sequencing data for *H. melpomene* male and female heads, antennae, legs, and mouth parts were obtained from ArrayExpress projects E-MTAB-1500 and E-MTAB-6249 (supplementary Table S1, Supplementary Material online). A four tissue *de novo* transcriptome made from one library per tissue type per sex was used as reference (see Macias-Muñoz et al. 2017). Reads from each sample were mapped to the transcriptome using bwa (Li and Durbin 2009) and RSEM (Li and Dewey 2011) was used to quantify mapped raw reads. We used edgeR (Robinson et al. 2010) to perform three pairwise comparisons for differential expression analysis: Heads versus antennae, heads versus legs, and heads versus mouth parts. For each comparison, a generalized linear model was used to include terms for batch, tissue, sex, the interaction of sex, and tissue (∼batch + tissue + sex + sex*tissue). Each analysis also included filtering to remove contigs with low expression (<1 count per million for at least four groups). Samples were normalized using a trimmed mean of the log expression ratios (TMM) (Robinson and Oshlack 2010). After each comparison, *P*-values were further corrected using a Bonferroni false discovery rate (FDR) correction. Contigs were considered significantly DE when the FDR was <0.05 and the log fold change (logFC) was >1. 

Of these DE contigs, we identified those which were upregulated in heads for each comparison. The resulting gene lists were merged to identify contigs commonly upregulated in heads. Patterns of expression for significant contigs and those commonly upregulated in heads were visualized using heatmaps (Ploner 2012). Contigs were annotated with *D. melanogaster* gene IDs (Marygold et al. 2012) by using command-line BLAST+ to compare *H. melpomene* transcriptome sequences to *D. melanogaster* gene sequences (Camacho et al. 2009). We used batch download in Flybase to acquire gene ontology (GO) terms for our DE and head upregulated contigs. DE contigs with unique annotations were enriched for function using a Database for Annotation, Visualization, and Integrated Discovery (DAVID) (Huang et al. 2009). Contigs upregulated in heads were also assigned GO terms and protein classification by NCBI BLAST and InterProScan in BLAST2GO to uncover additional annotations potentially missing from a comparison to *D. melanogaster* only (Conesa et al. 2005; Conesa and Götz 2008; Götz et al. 2008).

### Phototransduction Genes in Insect Genomes

To identify phototransduction genes in Lepidoptera and explore their evolutionary history, we used *D. melanogaster* sequences to search for homologs in published genomes. We began with a compilation of sequences by Bao and Friedrich (2009) but expanded it to include Lepidoptera species and additional phototransduction genes (supplementary Table S2, Supplementary Material online). We used BLAST to search the genomes of *Anopheles gambiae*, *Apis mellifera*, *Tribolium castaneum*, *B. mori*, *M. sexta*, *H. melpomene*, and *D. plexippus*. Sequences with identity of more than 20% and an E-value greater than 1E−10 were tested for homology using reciprocal blastp to the NCBI database. The search for *D. melanogaster* homologs in eight insect genomes resulted in a list of 76 unique genes from phototransduction gene families in insects. In addition to searching lepidopteran reference genomes, we searched *de novo* transcriptomes to improve annotations and find duplicates that are not found in genomes. We searched a *H. melpomene* four tissue transcriptome (Macias-Muñoz et al. 2017) and a *M. sexta* head transcriptome (Smith et al. 2014). We used Trinity to generate a *de novo* transcriptome using two *D. plexippus* adult whole heads. The *de novo* transcriptome was used in addition to the genome to confirm gene duplications (supplementary Tables S3–S5, Supplementary Material online). The nucleotide sequences recovered from *de novo* transcriptomes were translated using OrfPredictor with the blastx option before testing them by reciprocal blast hits (Min et al. 2005).

Sequence corrections were accomplished by aligning sequences in molecular evolutionary genetics analysis (MEGA) software and manually correcting missing pieces. BLAST was then used to recover the segment from the genome. To obtain the consensus sequences, we inputted corrected sequences to CLC Genomics (CLCBio) and mapped reads against them. With some exceptions, we recovered the entire sequence for all phototransduction genes in *H. melpomene* (supplementary Table S3, Supplementary Material online), *M. sexta* (supplementary Table S4, Supplementary Material online), and *D. plexipplus* (supplementary Table S5, Supplementary Material online). Phototransduction genes for *H. melpomene*, *M. sexta*, and *D. plexippus* were annotated and deposited in GenBank with accession numbers MK983015–MK983088, MK983089–MK983165, and MN037884–MN037955 (supplementary Tables S3–S5, Supplementary Material online). In addition, to examine the evolution of the *inaE* gene in *D. melanogaster* and the *DAGLβ*-like gene in lepidopterans in a wider context, we searched NCBI for insect sequence matches as well as matches to *Homo sapiens*, *Mus musculus*, and *Hydra vulgaris* sequences.

Protein sequences for each gene family were aligned in MEGA 7.0 using the Multiple Sequence Comparison by Log-Expectation (MUSCLE) algorithm (Edgar 2004; Kumar et al. 2016). The alignments were further corrected manually. Before generating maximum likelihood trees, we calculated Bayesian Information Criterion values to assess which substitution model would best fit our data (Schwarz 1978; Kumar et al. 2016). We used the best fit model to generate phylogenies using 100 bootstrap replicates (supplementary Table S6, Supplementary Material online).

### Expression of Candidate Genes

To study expression patterns among homologs, we looked at the expression of all genes found in 32 phototransduction gene families in *M. sexta* heads and in *H. melpomene* heads, antennae, legs, and mouth parts (i.e., labial palps+proboscis). Rearing conditions for *M. sexta* are described in Smith et al. (2014) and for *H. melpomene* in Briscoe et al. (2013) and Macias-Muñoz et al. (2017). We began by adding our corrected *H. melpomene* and *M. sexta* sequences (supplementary Tables S3 and S4, Supplementary Material online) to the *de novo* transcriptome assembly. We uniquely mapped trimmed and parsed reads from four male and four female *M. sexta* heads (E-MTAB-2066; Smith et al. 2014) to the corrected *M. sexta* transcriptome using bowtie v. 1.0 (Langmead et al. 2009). We also mapped processed reads from *H. melpomene* heads, antennae, legs, and mouth parts (E-MTAB-1500, E-MTAB-6249, E-MTAB-6342; Macias-Muñoz et al. 2017) to the corrected *H. melpomene* transcriptome. RSEM was used to count raw reads mapped (Li and Dewey 2011). We visualized expression levels by graphing *T*ranscripts *P*er *M*illion (TPM) for each gene of interest using ggplot2 (Wickham 2009). Differential expression between tissue types for *H. melpomene* was repeated as outlined above in edgeR using uniquely mapped reads to the transcriptome with corrected sequences. However, for this data set to allow for less stringency, we used *q*-values (Dabney and Storey 2013) to correct *P*-values rather than Bonferroni.

### Immunohistochemistry

An antibody was generated against the peptide N-CKGARTVDEDKKKE-C of the *H*. *melpomene* unclassified opsin (UnRh) in guinea pig and was immunoaffinity purified (New England Peptide, Gardner, MA, USA). We also used an antibody against the long-wavelength sensitive opsin (LWRh) of *Limenitis astyanax* (Frentiu et al. 2007; 2015) which labels LWRh expressing cells in *Heliconius* (McCulloch et al. 2016). Eyes were fixed, sucrose protected, cryosectioned, and immunolabeled according to methods in McCulloch et al. (2016). Following washes with 1× Phosphate-buffered saline and block (McCulloch et al. 2016; Macias-Muñoz et al. 2017), slides were incubated with 1:15 rabbit anti-LWRh and 1:30 guinea pig anti-UnRh antibodies in blocking solution overnight at 4°C. After washing in 1× Phosphate-buffered saline, slides were incubated with 1:500 goat anti-rabbit Alexafluor 555 and 1:250 goat anti-guinea pig Alexafluor 633 secondary antibodies in blocking solution for 2 h at room temperature in the dark. Slides were washed once more in 1× PBS in the dark and stored for imaging in Aqua Poly/Mount (Polysciences, Inc. Cat. No. 18606). Images were taken at the UC Irvine Optical Biology Core Facility using a Zeiss LSM700 confocal microscope under a 20× objective. Two-channel composites were generated using Fiji and brightness was adjusted for clarity using Adobe Photoshop.

## Results and Discussion

### Transcriptome-Wide Differential Expression Analysis

To determine the possible functions of genes expressed in butterfly heads, we used *H. melpomene* RNA-Seq data to identify contigs upregulated in head tissues relative to antennae, legs, and mouth parts. We predicted that head upregulated contigs would be annotated with GO terms associated with vision. A multidimensional scaling plot showed that head RNA-Seq profiles group together and away from other tissue types (supplementary fig. S1*A*, Supplementary Material online). Differential expression analysis comparing heads versus antennae yielded 1,173 DE contigs (supplementary fig. S2 and Table S7, Supplementary Material online), 561 of these were upregulated in heads (Table 1). Analysis of head versus legs mRNAs gave 1,472 DE contigs (supplementary fig. S2 and Table S8, Supplementary Material online). Of these contigs, 928 were upregulated in heads. Heads versus mouth parts comparison yielded 1,486 DE contigs (supplementary fig. S2 and Table S9, Supplementary Material online); 914 of these were upregulated in heads (Table 1). DE contigs from each of the three pairwise comparisons matched 576, 730, and 685 unique gene FlyBase gene IDs (Table 1).

**Table 1:** Summary of *Heliconius melpomene* Transcriptome-Wide Analysis

	Bonferroni	Upregulated in Heads	Commonly Upregulated in Heads	Unique FlyBase Gene ID
Head versus antennae	1,173	561		576
Head versus legs	1,472	928		730
Head versus mouth	1,486	914		685
Merged^a^			281	154

^a^Merged are genes commonly upregulated in heads after merging results of pairwise comparisons.

Most of the genes enriched in the DE analyses between heads and other tissues have vision-associated functions (supplementary Results and Table S10, Supplementary Material online), as has been found in a transcriptomic analysis of *M. sexta* adult head tissue alone (Smith et al. 2014). This could be because more transcription is actively occurring in the adult butterfly head and the head is mostly composed of the eye and optic lobe (Girardot et al. 2006). *Heliconius* butterflies have large eyes due to selective pressures that favor development of big eyes relative to body size. The optic lobe accounts for ∼64% of the total brain volume (Seymoure et al. 2015; Montgomery et al. 2016).

### Head Upregulated Genes

We merged the lists of contigs upregulated in heads in each pairwise comparison to obtain 281 contigs commonly upregulated in heads across the three comparisons (Table 1). Head upregulated contigs annotated using BLAST2GO level 2 analysis showed that 78 of the annotated genes were involved in cellular processes and 32 were involved in responses to stimulus (supplementary fig. S1*C*, Supplementary Material online) (Conesa et al. 2005; Conesa and Götz 2008; Götz et al. 2008, 2011). A multilevel analysis of all head upregulated contigs shows that ∼33% are involved in ion transmembrane transport and 23% in G protein coupled receptor signaling pathways (supplementary fig. S1*D*, Supplementary Material online).

The 281 commonly upregulated and annotated contigs in heads across the three comparisons corresponded to 154 unique *D. melanogaster* FlyBase gene IDs (Table 1; supplementary Table S11, Supplementary Material online). These 154 contigs were grouped into eleven annotation clusters using the highest stringency in DAVID (supplementary fig. S1E; Huang et al. 2009). The top three annotation clusters were: 1) detection of light stimulus, 2) regulation of rhodopsin-mediated signaling pathway, and 3) detection of light stimulus involved in visual perception (supplementary fig. S1*E*, Supplementary Material online). The genes grouped within these clusters were annotated with phototransduction functions due to homology with *D. melanogaster* genes, *Rh3*, *Rh5*, *Gbeta76*, *norpA*, *ninaC*, *ninaA*, *INAD*, *Calx*, *trpl*, *Arr1*, *Arr2*, and *stops* (further discussed below; supplementary fig. S1*E*, Supplementary Material online). Of the remaining eight annotation clusters, clusters 9 and 10 are also directly associated with vision and are enriched for homeobox and rhabdomere development, respectively. Two genes in common between these two clusters include *PvuII-PstI homology 13* (*Pph13*) and *ocelliless* (*oc*) that function in ocellus and compound eye photoreceptor development (Fichelson et al. 2012; Mahato et al. 2014).

Some of the genes enriched in other annotation clusters also have a role in vision. One gene in common between annotation clusters 4, 5, and 6 is *ora transientless* (*ort*), a gene that is necessary for vision as it encodes a postsynaptic chlorine channel gated by the photoreceptor neurotransmitter, histamine (Gengs et al. 2002). Annotation clusters 4, 5, and 8 include *resistant to dieldrin* (*Rdl*), a gene that has a role in the circuits underlying visual processing, odor coding, learning and memory, sleep, and courtship behavior (Brotz et al. 2001; Liu et al. 2007; Chung et al. 2009; Yuan et al. 2014).

### Conservation of Phototransduction Genes in Lepidoptera

**Fig. 2.**
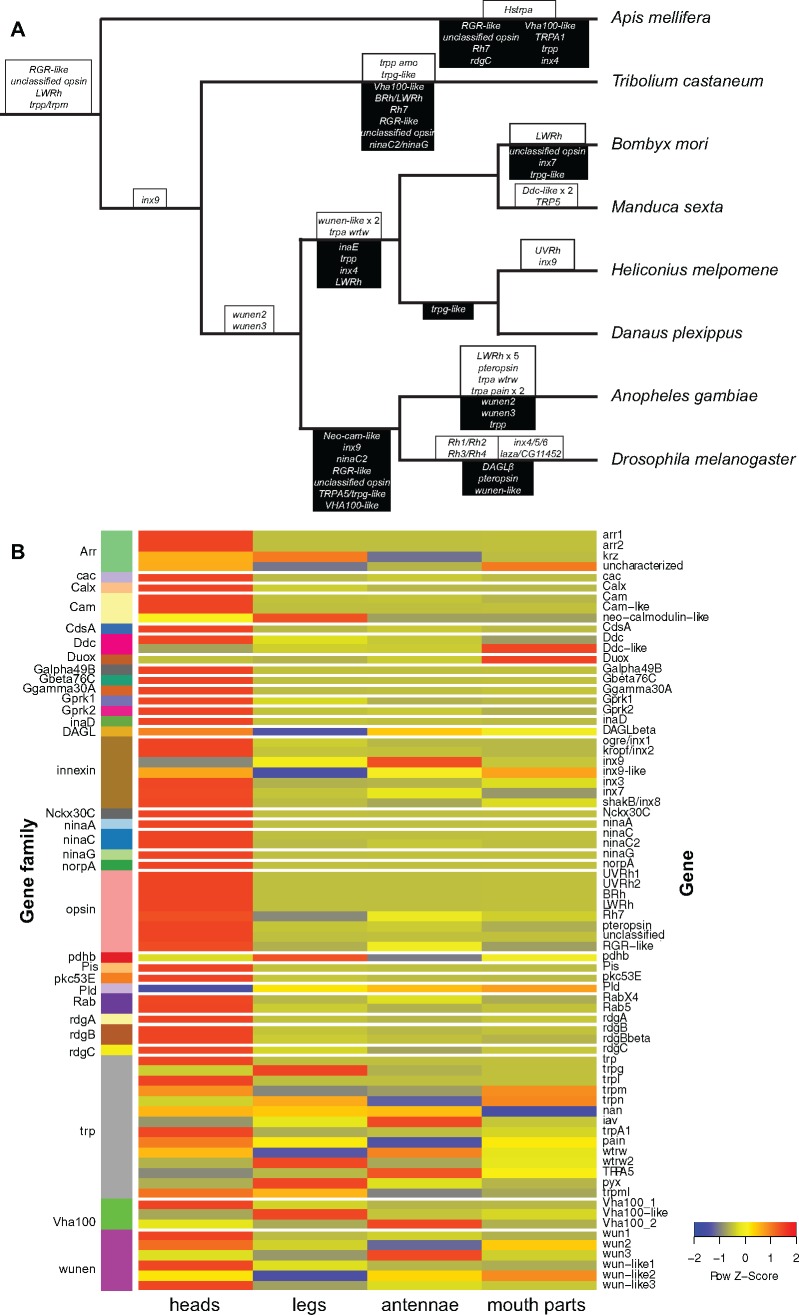
—Phototransduction gene gains, losses, and expression. Most changes across the insect phylogeny occur in the *opsin* and *trp* gene families. (*A*) Insect phylogeny showing gains in white boxes above branches and losses in black boxes below branches. (*B*) Heatmap of expression of genes orthologous to *D. melanogaster* phototransduction genes in *Heliconius melpomene* heads, antennae, legs, and mouth parts. Red signifies high expression while blue signifies low expression. Gene names are listed on the right while gene family names are listed on the left and assigned a different block color per gene family. Most vision-related genes have elevated expression in the butterfly head.

Genes commonly upregulated in *H. melpomene* heads were annotated with functions relating to vision and phototransduction in *Drosophila* (supplementary fig. S1, Supplementary Material online). Yet their evolutionary history and potential functional conservation requires further validation. To evaluate whether phototransduction genes were lost or expanded in Lepidoptera relative to *D. melanogaster*, we generated 32 insect phylogenies for 76 phototransduction-related genes (supplementary Tables S2–S5, Supplementary Material online). For each phylogeny, we searched eight insect genomes including two moth species (*M. sexta* and *B. mori*) and two butterfly species (*H. melpomene* and *D. plexippus*). Across all eight insect genomes we detected gene gains and losses in gene families such as *opsin*, *trp*, *innexin*, and *lazaro/wunen* (fig. 2*A*). Between *D. melanogaster* and lepidopterans, differences in gene gain and loss occur in the gene families *opsin*, *innexin*, *lazaro/wunen*, and *DAGL*. We did not detect any conserved differences in gene gain or loss between moths and butterflies (fig. 2*A*). Yet, an interesting gene family to note is *Vha100*, which has a *Vha100-like* gene that is lost in nonlepidopteran insects (supplementary Results and fig. S6*G*, Supplementary Material online) and also *innexin 9*, which is duplicated in *H. melpomene* (supplementary Results and fig. S7, Supplementary Material online).

Because many genes seem to be conserved between *D. melanogaster* and Lepidoptera, we visualized their expression in *H. melpomene* heads, antennae, legs, and mouth parts. Upregulation of orthologs in *H. melpomene* heads would suggest a conserved role in vision for genes annotated with phototransduction function. Conversely, upregulation of a paralog suggests that butterflies are using a different member of the gene family to perform a visual function. We found 32 genes upregulated in heads relative to other tissue types (fig. 2*B*;Table 2; supplementary figs. S3–S7, Supplementary Material online). Most of the main genes involved in *D. melanogaster* phototransduction were found as single copies in Lepidoptera and were upregulated in *H. melpomene* heads such as *Gqα*, *β* and *γ*, *norpA*, *inaD*, *ninaC*, *Calx*, *trp*, *trpl*, *Arr1*, *Arr2*, and *stops* (fig. 1; Table 2, for additional orthocluster analysis in butterflies see Catalán et al. 2018). These results suggest that second messengers, ion channels, and termination of phototransduction are conserved between *D. melanogaster* and Lepidoptera (see below). The main differences in the phototransduction cascade between *H. melpomene* and *D. melanogaster* are in the opsins which initiate phototransduction and in DAG regulation (discussed further below; fig. 1). Although there is no consistent difference between moths and butterflies in gene gains and losses, we found large differences in *trp* gene expression (see below).

**Table 2:** *Q*-Values for Four Tissue Pair-Wise Comparisons in *Heliconius melpomene*

Gene Family	Gene Symbol	Head Versus Antennae	Head Versus Legs	Head Versus Mouth
Arr	*Arr2*	**2.33E−15**	**1.33E−32**	**2.03E−19**
Arr	*Arr1*	**2.56E−11**	**1.16E−12**	**5.22E−14**
Arr	*krz*	0.467	**0.023**	0.061
Arr	*uncharacterized*	0.192	**1.60E−04**	0.455
cac	*cac*	0.225	**1.81E−04**	**1.07E−09**
Calx	*Calx*	**6.76E−04**	**8.48E−10**	**5.46E−07**
Cam	*Cam-like*	**1.99E−11**	**1.24E−18**	**1.96E−16**
Cam	*neo-calmodulin-like*	**0.003**	**0.003**	**7.63E−07**
Cam	*Cam*	**0.043**	**1.39E−04**	**0.005**
CdsA	*CdsA*	**0.003**	**7.53E−18**	**5.21E−14**
DAGL	*DAGLbeta*	**2.37E−04**	0.489	0.409
Ddc	*Ddc-like*	**1.20E−05**	**1.46E−06**	**4.16E−13**
Ddc	*Ddc*	0.132	0.240	0.278
Duox	*Duox*	**0.028**	**0.009**	**9.77E−07**
Galpha49B	*Galpha49B*	**7.24E−25**	**3.51E−34**	**2.87E−22**
Gbeta76C	*Gbeta76C*	**5.3E−20**	**1.69E−20**	**5.55E−20**
Ggamma30A	*Ggamma30A*	**2.83E−05**	**5.89E−10**	**7.20E−07**
Gprk1	*Gprk1*	0.176	0.146	0.097
Gprk2	*Gprk2*	0.544	0.093	0.124
inaD	*inaD*	**1.40E−07**	**3.40E−07**	**1.68E−07**
innexin	*shakB/inx8*	**8.05E−10**	**2.69E−10**	**0.003**
innexin	*ogre/inx1*	0.064	0.061	**0.021**
innexin	*kropf/inx2*	0.070	0.061	**0.037**
innexin	*inx9-like*	N/A	N/A	N/A
innexin	*inx9*	0.038	0.142	0.273
innexin	*inx3*	0.355	0.425	0.416
innexin	*inx7*	N/A	N/A	N/A
Nckx30C	*Nckx30C*	**0.007**	**3.75E−09**	**1.16E−10**
ninaA	*ninaA*	**8.24E−16**	**4.54E−18**	**1.72E−17**
ninaC	*ninaC*	**1.18E−25**	**4.57E−29**	**7.34E−27**
ninaC	*ninaC2*	**7.7E−09**	**7.71E−16**	**9.16E−16**
ninaG	*ninaG*	**1.22E−12**	**8.15E−20**	**8.72E−14**
norpA	*norpA*	**4.55E−24**	**5.01E−22**	**1.82E−18**
opsin	*LWRh*	**4.1E−21**	**3.54E−27**	**5.63E−26**
opsin	*BRh*	**3.9E−28**	**1.77E−22**	**3.41E−20**
opsin	*unclassified*	**2.13E−17**	**1.86E−18**	**6.64E−12**
opsin	*UVRh1*	**1.62E−14**	**7.53E−18**	**3.17E−09**
opsin	*UVRh2*	**1.28E−09**	**1.48E−06**	**1.78E−07**
opsin	*RGR-like*	**0.022**	0.159	0.058
opsin	*Rh7*	0.610	0.276	0.244
opsin	*pteropsin*	N/A	N/A	0.124
Pdhb	*Pdhb*	0.374	**0.007**	0.439
Pis	*Pis*	**0.002**	**6.47E−09**	**3.17E−10**
pkc53E	*pkc53E*	**2.19E−07**	**1.40E−07**	**3.17E−09**
Pld	*Pld*	0.167	**0.020**	**0.044**
Rab	*Rax4*	0.232	**7.75E−04**	**1.95E−06**
Rab	*Rab5*	0.267	0.035	0.166
rdgA	*rdgA*	**4.06E−04**	**0.001**	**0.002**
rdgB	*rdgB*	**1.07E−04**	**1.97E−08**	**1.40E−04**
rdgB	*rdgBbeta*	0.488	0.127	0.060
rdgC	*rdgC*	0.083	0.024	0.248
trp	*trpl*	**8.17E−20**	**6.25E−24**	**6.21E−19**
trp	*TRPA5*	**4.64E−21**	**3.41E−08**	**2.29E−12**
trp	*trp*	**7.05E−05**	**2.96E−05**	**9.93E−06**
trp	*TrpA1*	0.200	**0.002**	**0.002**
trp	*nan*	0.479	0.469	**0.002**
trp	*Trpm*	0.350	0.384	**0.047**
trp	*trpn*	0.514	**0.039**	0.166
trp	*trpml*	0.190	0.370	0.204
trp	*pain*	0.437	0.143	0.217
trp	*trpg*	0.565	0.364	0.297
trp	*iav*	**2.82E−04**	0.089	0.343
trp	*wtrw*	0.455	0.420	0.490
trp	*pyx*	N/A	**1.98E−04**	N/A
trp	*wtrw2*	N/A	N/A	N/A
Vha100	*Vha100-1*	0.146	**0.010**	**0.011**
Vha100	*Vha100-like*	0.187	**2.51E−04**	0.085
Vha100	*Vha100-2*	**2.45E−05**	**0.045**	0.119
wunen	*wun-like3*	0.062	**0.002**	**0.011**
wunen	*wun1*	0.365	0.104	**0.013**
wunen	*wun3*	0.211	0.466	0.307
wunen	*wun-like1*	0.299	0.288	0.310
wunen	*wun-like2*	0.582	0.307	0.331
wunen	*wun2*	**0.001**	0.159	0.473

Bold numbers represent significance at a level of *P*-value <0.05.

### Opsins in Lepidoptera

**Fig. 3.**
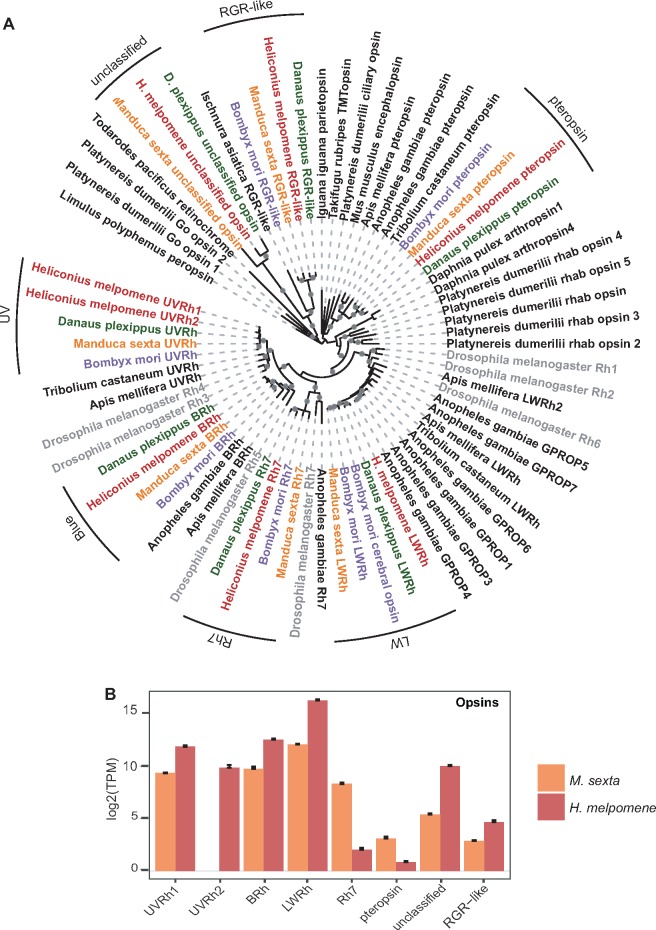
—Insect opsin phylogeny and opsin gene expression in a moth and butterfly. Lepidopterans have two opsin genes related to squid retinochrome, *unclassified* and *RGR-like*, not found in *D. melanogaster.* (*A*) Opsin phylogenetic tree generated using amino acid sequences from Kanost et al. (2016) and from *Heliconius melpomene* and *Danaus plexippus*. *D. melanogaster* is in gray while lepidopteran species are in different colors, *Bombyx mori* (purple), *Manduca sexta* (orange), *D. plexippus* (green), and *H. melpomene* (red). Maximum-likelihood tree was generated using opsin amino acid sequences from 17 species with an LG+G + I+F model. Lepidopteran opsin clades are indicated by black labeled arcs. (*B*) Expression of opsin genes in *M. sexta* heads (*n* = 8, orange) and *H. melpomene* heads (*n* = 8, red) measured using RNA-Seq. The *y* axis is in transcripts per million on a log2 scale. Bars indicate standard errors.

We began our survey of phototransduction genes in Lepidoptera by investigating the molecular evolution and expression of opsin genes typically responsible for initiating the phototransduction cascade (fig. 1). To inspect the phylogenetic history of the opsins, we added *H. melpomene* sequences from the reference genome and a four-tissue *de novo* transcriptome (Macias-Muñoz et al. 2017) to a set of sequences used in Kanost et al. (2016). We recovered the previously described *Heliconius-*specific *UVRh* duplication and orthologs for all other known opsins (fig. 3*A*) (Briscoe et al. 2010; Yuan et al. 2010; McCulloch et al. 2017). We also found two opsin genes: An *unclassified opsin* (*UnRh)* first described in Kanost et al. (2016) and *RGR-like* that both lack a *D. melanogaster* ortholog but are found in our butterfly genomes (fig. 3*A*).

To determine a role for all opsin genes we looked at their expression profile in *M. sexta* and *H. melpomene.* We expected opsins involved in vision to be highly expressed in heads. In *M. sexta*, all opsins had expression in head tissue (fig. 3*B*). In *H. melpomene*, our functional enrichment showed that homologs of *D. melanogaster* rhodopsin genes *Rhodopsin 3* (*Rh3*) and *Rhodopsin 5* (*Rh5*), which correspond to *UVRh1/Rh2* and *BRh*, respectively, were upregulated in *H. melpomene* heads (supplementary fig. S1*E* and Table S11, Supplementary Material online) (Briscoe et al. 2010; Yuan et al. 2010). *LWRh* and the *unclassified opsin* (*UnRh*) are also upregulated in *H. melpomene* heads (Table 2; fig. 3*B*). *LWRh* was the most highly expressed opsin gene probably due to the amount of LW photoreceptor cells per ommatidium. *Heliconius* ommatidia have nine photoreceptor cells each where at least six cells express LWRh and two express short wavelength BRh, UVRh1, or UVRh2 (McCulloch et al. 2016, 2017).

Upregulation of *UnRh* was intriguing because Kanost et al. (2016) noted the unclassified opsin lacks a lysine at the typical location where the chromophore necessary to initiate phototransduction is bound in opsins, yet the gene is highly expressed in *H. melpomene* eyes and brain suggesting a role in vision (fig. 3; Table 2). A recent study found that alternative amino acid sites may be used in some G-protein coupled receptors for chromophore-binding (Faggionato and Serb 2017). Furthermore, cephalopods have a photosensitive pigment called retinochrome, studied biochemically, that lacks a conserved rhodopsin glutamic acid base (Terakita et al. 1989, 2000). Retinochrome, unlike rhodopsin, binds an all-*trans* retinal and acts as a photoisomerase converting the chromophore to 11-*cis* to regenerate the photosensitive rhodopsin (Sperling and Hubbard 1975). By adding a squid retinochrome sequence to our opsin phylogeny we found that the lepidopteran-specific unclassified opsin and RGR-like opsin are more closely related to retinochrome than they are to other opsins with known functions (fig. 3*A*).

**Fig. 4.**
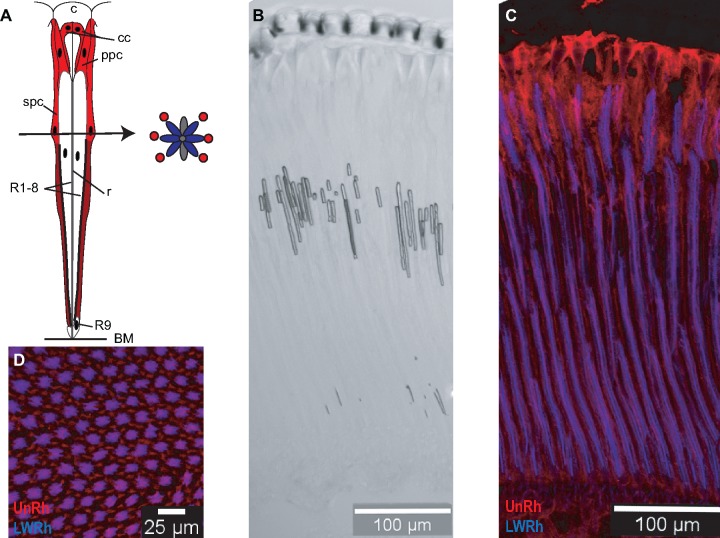
—Immunohistochemistry of a butterfly retinochrome, unclassified opsin (UnRh). UnRh is expressed in several kinds of cells found in the distal retina but not in photoreceptor cells. (*A*) Drawing of a butterfly ommatidium showing the cornea (c), crystalline cone (cc), rhabdom (r), photoreceptor cells (R1-9), primary pigment cells (ppc), secondary pigment cells (spc), and basement membrane (bm) based on Kolb (1985). Red represents areas where UnRh expression is detected, dark red indicates where the cell presumably narrows and staining is not as bright. A drawing of a cross section shows cells R1-8, blue cells represent LWRh staining and red circles represent UnRh staining. (*B*) Brightfield image of a longitudinal section of a *Heliconius melpomene* eye showing the anatomy of each ommatidium and an intact cornea. (*C*) Fluorescent image of the same section stained for opsins using rabbit anti-LWRh (blue) and guinea pig anti-UnRh (red) antibodies. (*D*) Transverse section stained for opsins LWRh (blue) and UnRh (red).

As *UnRh* has high expression in eyes and is phylogenetically similar to retinochrome, both proteins may have related enzymatic roles in vision if UnRh is expressed near the photoreceptor cells. To localize where in the butterfly eye UnRh is expressed, we made an antibody against one of its unique domains. We visualized UnRh expression alongside that of LWRh in *H. melpomene*. In *Heliconius*, LWRh is expressed in photoreceptor cells R3-8 (McCulloch et al. 2016). Intriguingly, we found UnRh abundantly expressed in crystalline cone cells, in primary pigment cells, and in the six secondary pigment cells surrounding the ommatidium (fig. 4). Staining is brighter in the distal part of the retina presumably because the secondary pigment cells decrease in size as they approach the basement membrane (fig. 4*A*). If UnRh had a function similar to that of the color vision opsins, we would expect it to be expressed in the photoreceptor cells. However, this protein is expressed in other retina cells adjacent to the photoreceptor cells. In squid, retinochrome is expressed in inner segment cells while the rhodopsin that it interchanges chromophore with is in the outer segment, separated by the basement membrane (Kingston et al. 2015; Chung and Marshall 2017). Taken together, these results suggest that UnRh might have a role similar to squid retinochrome in photoisomerization of the butterfly chromophore. This mechanism could be required for fast regeneration of an active rhodopsin necessary to quickly process visual information during flight.

In flies, the presence of 11-*cis* 3-hydroxyretinal is necessary for the synthesis of rhodopsin, suggesting a mechanism needs to be in place to rapidly convert the all-*trans* form into a reactive molecule. In *Drosophila*, all-*trans* 3-hydroxyretinal is transported to the pigment cells where a photoisomerase converts it back into the 11-*cis* configuration by blue light (Stavenga et al. 2017). Light intensity and wavelength affect the rate of 11-*cis* 3-hydroxyretinal synthesis in blowflies meaning that photoregeneration maintains levels of rhodopsin (Schwemer 1984). Interestingly, Lepidoptera are thought to rely more on enzymatic regeneration of 11-*cis* 3-hydroxyretinal than is the case in Diptera (Bernard 1983a, 1983b; Stavenga and Hardie 2011). Furthermore, a retinal-binding protein (RBP) was found in honeybees that binds the all-*trans* retinal that is isomerized in light (Pepe and Cugnoli 1980). Studies of honey bee RBP-A and RBP-B found that RBP-B binds all-*trans* retinal and also catalyzes the photoisomerization into the 11-*cis* conformation (Schwemer et al. 1984). Honey bee RBP-B function is similar to squid retinochrome but is unlikely to be a member of the same gene family due to its size (Pepe and Cugnoli 1980). Like these proteins, UnRh in butterflies may also have the ability to photoisomerize the chromophore molecule.

### Regulation of DAG

**Fig. 5.**
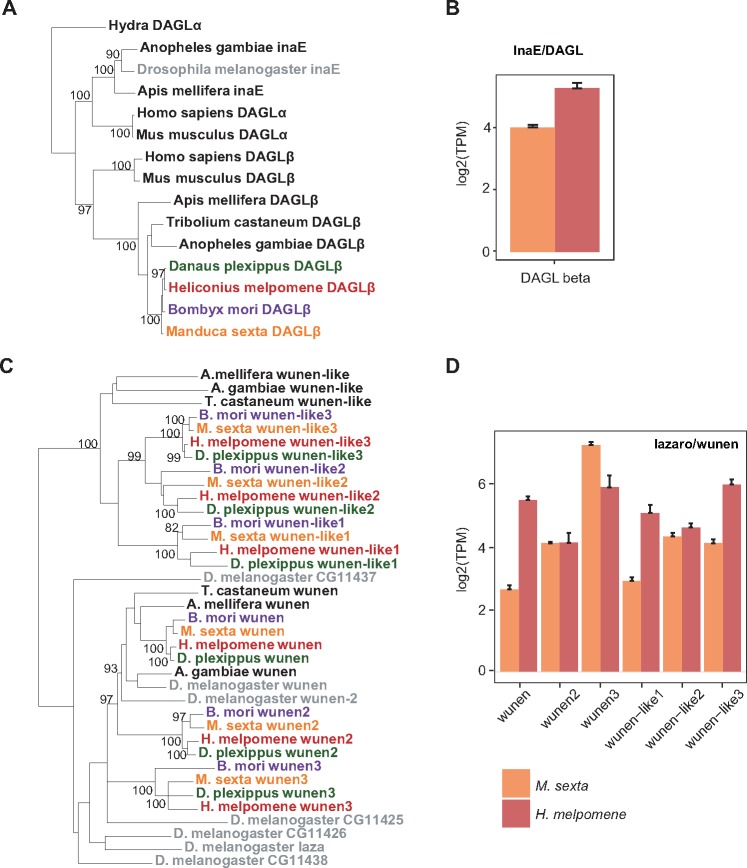
—Molecular evolution and expression of DAGL and lazaro/wunen. In lepidopterans, orthologs of *Drosophila melanogaster DAGLα* and *lazaro* are missing. Other gene family members may be playing a similar role in lepidopterans. (*A*) DAGL phylogenetic tree generated using amino acid sequences from eight insect genomes and *Homo sapiens* and *M. musculus.* Phylogenetic label colors follow those of figure 3. (*B*) Expression of *DAGL* genes in *Manduca sexta* heads (*n* = 8) and *Heliconius melpomene* heads (*n* = 8). (*C*) Wunen phylogenetic tree generated using amino acid sequences from eight insect genomes. (*D*) Expression of *wunen* genes in *M. sexta* heads (*n* = 8, orange) and *H. melpomene* heads (*n* = 8, red) measured using RNA-Seq. The *y* axis is in transcripts per million on a log2 scale. Bars indicate standard errors.

After phototransduction is triggered by photon absorption, G*α*q is released from a G-protein complex of three subunits (*α*, *β*, and *γ*) and activates PLC (is encoded by *norpA*) to produces DAG (Bloomquist et al. 1988; Lee et al. 1994). DAG has been implicated in the activation of TRP and TRPL channels (Chyb et al. 1999; Leung et al. 2008). DAG is hydrolyzed by the actions of DAG lipase (DAGL) encoded by the gene *inaE* (Leung et al. 2008). *InaE* mutants in *D. melanogaster* have defective responses to light, demonstrating that DAGL activity is required for photoreceptor responses (Leung et al. 2008). Although this gene is crucial for *D. melanogaster* phototransduction, an ortholog of *inaE* is missing in Lepidoptera (fig. 2*A*;supplementary Table S2, Supplementary Material online). We found that Lepidoptera retains *DAGLβ*, *D. melanogaster* retains *DAGLα* (*inaE*), and *A. mellifera*, *A. gambiae, T. castaneum*, and mammals retain both (fig. 5*A*). Both *DAGLα* and *DAGLβ* encode an Sn-1 DAGL that generates a monoacylglycerol (MAG) product. Note that for *T. castaneum*, *DAGLα* is not included in the phylogeny because the sequence was too short to generate a correct alignment. We predict that *DAGLβ* carries out the phototransduction function of hydrolyzing DAG in moth and butterfly vision because Lepidoptera have lost an ortholog of *D. melanogaster inaE* and have retained *DAGLβ*. *DALGβ* was expressed in *M. sexta* heads and in *H. melpomene* heads (fig. 5*B*). Although we confirm expression in heads, *DAGLβ* is not upregulated in heads relative to other tissue types. *DAGLβ* may have a role in vision in Lepidoptera, but it might also be used in other tissues for other functions. The role of *DAGLβ* in other insects is not clear. However, in humans and mice *DAGLα* and *DAGLβ* are necessary for axonal growth and synaptic signaling and inhibiting these proteins results in changes in brain signaling (Bisogno et al. 2003; Ogasawara et al. 2016). Interestingly, although both *DAGLα* and *DAGLβ* are expressed in axonal tracts and the developing spinal cord, only *DAGLβ* is expressed in the retinal ganglion layer and the optic lobe (Bisogno et al. 2003).

DAG level is also regulated by degeneration A (RDGA) (conserved in moths and butterflies; supplementary fig. S6, Supplementary Material online) and Lazaro (LAZA) (Garcia-Murillas et al. 2006; Bao and Friedrich 2009). Lazaro is a lipid phosphate phosphatase and is found in *D. melanogaster* photoreceptors (Garcia-Murillas et al. 2006). *Lazaro* is a member of the *wunen* subfamily (fig. 5*C*). *Wunen* helps regulate the level of bioactive phospholipids, has a role in germ line migration and is necessary for tracheal development (Zhang et al. 1997; Ile et al. 2012). We found seven sequences belonging to the *wunen* gene family in *D. melanogaster*; *Lazaro* is a *D. melanogaster-*specific duplication, as previously noted (Bao and Friedrich 2009). Although other non-*D. melanogaster* insects have one copy of *wunen*, lepidopterans have three copies (fig. 5*C*). In addition, although other insects have one copy of *wunen-like*, Lepidoptera have three copies of *wunen-like* that arose after lepidopteran divergence from other insects (fig. 5*C*). All copies of *wunen* and *wunen-like* are expressed in *M. sexta* and *H. melpomene* heads (fig. 5*D*). *Wunen* and *wunen-like3* are the two copies most highly expressed in *H. melpomene* heads. Taken together, the above results suggest a difference in the gene family members involved in DAG regulation between *D. melanogaster* and lepidopterans.

### TRP Channels

TRP and TRPL channels are essential in *D. melanogaster* phototransduction. They allow the influx of Ca^2+^ and cause cell depolarization (Montell and Rubin 1989). *Trp* is the dominant light-sensitive channel in *Drosophila* rhabdomeres (∼10× more abundant than *trpl*), and flies with mutated *trp* behave as though they are blind (Montell and Rubin 1989). The TRP superfamily contains more than 20 cation channels (Montell et al. 2002). Although *trp* and *trpl* function in *D. melanogaster* vision, other *trp* genes sense pain, vanilloid compounds, and heat, among other stimuli (Montell et al. 2002; Montell 2005). In our examination of the TRP gene family, we found 14 members in *H. melpomene* and 17 in *M. sexta*.

**Fig. 6.**
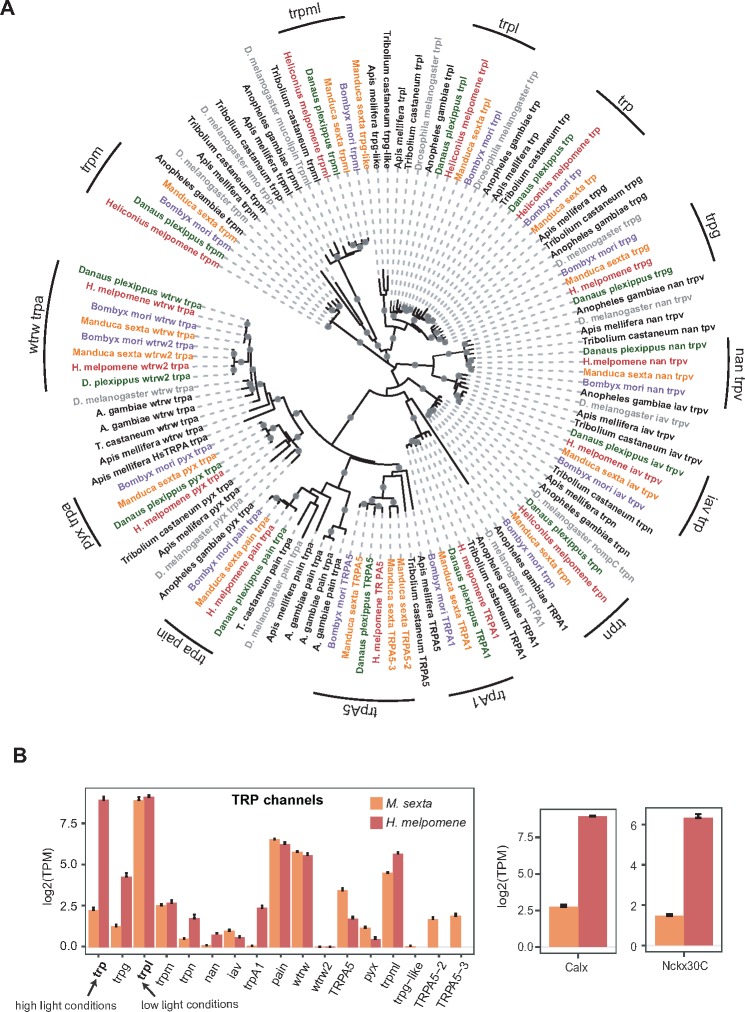
—Phylogeny and expression of the transient receptor potential (trp) cation channel gene family. Lepidopterans have a duplication of the *wtrw trpa* gene and *Manduca sexta* has a duplication of *TRPA5.* Expression of *trp* is ∼50× less than *trpl* in *M. sexta*, a lepidopteran which is active under dim light conditions. (*A*) *Trp* phylogenetic tree generated using amino acid sequences inferred from eight insect genomes using a WAG+G + F model*.* Lepidopteran trp clades are indicated by black labeled arcs. Phylogenetic label colors follow those of figure 3. (*B*) Expression of *trp*, *Calx*, and *Nckx30C* genes in *M. sexta* heads (*n* = 8) and *Heliconius melpomene* heads (*n* = 8) measured using RNA-Seq. The *y* axis is in transcripts per million on a log2 scale. Bars indicate standard errors. *Trp* and *trpl* shown in bold are used in high light and low light conditions, respectively, indicated by arrows.

Differences between *D. melanogaster* and Lepidoptera include a duplication of *trpa wtrw* and a loss of *trpp* in moths and butterflies. The function of *trpa wtrw* (encoding TRP channel water witch) has not been characterized in any insect species but *trpa* genes, related gene family members, have been shown to function in temperature sensitivity, fructose aversion, and sexual receptivity in *D. melanogaster* (Xu et al. 2008; Sakai et al. 2009; Peng et al. 2016). *Trpa wtrw* is expressed in *M. sexta* heads and in *H. melpomene* heads whereas *trpa wtrw2* has very low expression. In the *trp* family, *M. sexta* retains a *trpg-like* gene that is lost in *D. melanogaster* and butterflies *H. melpomene* and *D. plexippus* (figs. 2*A* and 6). *Trpg* encodes a protein that is found in *D. melanogaster* photoreceptors and has been speculated to form a heteromultimeric channel with TRPL (Montell 2005). The role of *trpg* in *Drosophila* vision is uncertain. It is expressed in *H. melpomene* heads, but *trpg* and *trpg-like* have low expression in *M. sexta* heads (fig. 6*B*). Furthermore, *M. sexta* also has three *TRPA5* genes*.* Other lepidopterans have one copy. *D. melanogaster* and *A. gambiae* do not have any copies (fig. 6*A*). All three *TRPA5* genes are expressed in *M. sexta* heads as is *TRPA5* in *H. melpomene* heads (fig. 6*B*).

### Ion Channels Used in Diurnal and Nocturnal Insects

A transcriptome study in cockroaches found that *trpl* was ∼10 times more abundant than *trp* (French et al. 2015). RNAi of *trpl* reduced electroretinogram responses much more than RNAi of *trp* after 21 days suggesting that, as opposed to *D. melanogaster*, cockroach TRPL rather than TRP has a larger contribution to phototransduction (French et al. 2015). The authors suggested that differences in visual ecology are responsible for differential functions of the ion channels: Daylight-active *D. melanogaster* rely on fast responsive TRP and dark- or dim-light active cockroaches rely on TRPL (French et al. 2015). We found that *trp* and *trpl* are both highly expressed in *H. melpomene* heads which is different from either *D. melanogaster* or cockroach. Like cockroaches, we found that *trp* and *trpl* both have expression in *M. sexta* heads, but *trp* is expressed at a much lower level compared with *trpl* (fig. 6*B*). Our results suggest that the TRPL ion channel is also used by Lepidoptera in low light conditions.

TRP and TRPL channels allow Ca^2+^ and Na^+^ into the photoreceptor cell and are co-localized with a Na^+^/Ca^2+^ exchanger encoded by *Calx* which allows Ca^2+^ out of the cell (Montell 2005). Mutations of *Calx* result in a transient light response and a decrease in signal amplification implying a role for this gene in Ca^2+^ maintenance for proper TRP signaling (Wang et al. 2005). Overexpression of *Calx* can suppress retinal degeneration due to TRP constitutive activation (Montell 2005; Wang et al. 2005). *Calx* is upregulated in *H. melpomene* heads and is found as a single copy in all insect genomes (fig. 6*B*;Table 2; supplementary fig. S3*C*, Supplementary Material online). We detected a lower expression of *Calx* in *M. sexta* heads compared with the expression in *H. melpomene* heads potentially correlated with the lower expression of *trp* compared with *trpl* in *M. sexta* (supplementary fig. S3*C*, Supplementary Material online). A similar pattern of expression was also observed for another Na^+^/Ca^2+^ exchanger encoded by *Nckx30C*. *Nckx30C* was upregulated in *H. melpomene* heads yet expression of this ion channel was lower in *M. sexta* heads compared with *H. melpomene* heads (Table 2; fig. 6*B*;supplementary fig. S5*B*, Supplementary Material online). *Nckx30C* has a similar role to *Calx* in moving Ca^2+^ out of the cell (Haug-Collet et al. 1999). Both *Nckx30C* and *Calx* are expressed in the embryonic nervous system of *D. melanogaster* and in the adult eye and brain (Haug-Collet et al. 1999). Our results suggest that decreased expression of *trp* in nocturnal moths is accompanied by a decrease in *Calx* and *Nckx30C* expression. We conclude that one difference between moth and butterfly phototransduction is in the expression of ion channels used for calcium exchange.

### Proposed Phototransduction Cascade in Lepidoptera

Based on phylogenetic relationships and gene expression analyses we propose a model of phototransduction in Lepidoptera (fig. 1). Phototransduction initiation requires an opsin to be bound to a chromophore to initiate the cascade. We propose that in Lepidoptera, the chromophore is transported by CTD31 rather than the ortholog of *D. melanogaster* PINTA, which has been lost in lepidopterans (Macias-Muñoz et al. 2017). Similar to *D. melanogaster*, visual opsins (*BRh*, *LWRh*, and *UVRh*) initiate the phototransduction cascade by a change in conformation when the chromophore molecule absorbs light energy (von Lintig et al. 2010). We note that lepidopterans vary in opsin number (Frentiu et al. 2007; Briscoe 2008; Pirih et al. 2010; Xu et al. 2013). Photoisomerized 11-*cis*-3-hydroxyretinal is supplied to light-activated rhodopsin by retinochrome (UnRh) proteins found in pigment cells. Change in opsin conformation due to light absorption triggers the G-protein signaling cascade. Gαq, β, and γ are present as single copies and highly expressed in heads suggesting a conserved function in PLC activation, encoded by *norpA*, when G*α*q is released (supplementary figs. S1*E*, S4*D*–S4*F*, and S5*E*, Supplementary Material online) (Bloomquist et al. 1988; Lee et al. 1994).

PLC produces InsP_3_ and DAG (Bloomquist et al. 1988; Hardie 2001). However, the regulation of DAG levels appears to differ between lepidopterans and *D. melanogaster* due to the absence of *laza* and the loss of *inaE*. We propose that in Lepidoptera the actions of *inaE* are undertaken by a lepidopteran paralog *DAGLβ* and those of *laza* by other members of the gene family, *wunen* or *wunen-like3*. LAZA acts in opposition to DAG kinase encoded by *rdgA* (Garcia-Murillas et al. 2006). In *D. melanogaster*, DAG is converted into PIP_2_ by the phosphoinositide pathway which gives photoreceptor cells sensitivity and fast response (Hardie 2001; Garcia-Murillas et al. 2006). The actions of this pathway seem conserved in Lepidoptera because *rdgA*, *cdsA*, and *rdgB* are upregulated in *H. melpomene* heads. Although phosphatidic acid (PA) is likely converted into DAG by a *laza* paralog (*wunen* or *wunen-like3*), kinase *rgdA* maintains a role in converting DAG into PA. *CDP-diacylglycerol synthase* encodes a protein that converts PA into cytidine diphosphate DAG (CDP-DAG). Phosphatidyl inositol (PI) synthase then changes CDP-DAG into PI which is transported by phosphatidylinositol transfer protein encoded by *rdgB*. Phosphorylation converts PI into PIP_2_. The actions by which DAG functions in phototransduction are not well understood. DAGL produces the metabolite polyunsaturated MAG (Montell 2012). DAG might activate TRP and TRPL channels, although its role in phototransduction is debated (Chyb et al. 1999; Leung et al. 2008).

TRP and TRPL allow Ca^2+^ and Na^+^ into the cell that causes the photoreceptor cell to depolarize (Montell and Rubin 1989). We propose that the phototransduction cascade varies between moths and butterflies in the deployment of TRP and Na^+^/Ca^+^ channels. According to our expression data, butterflies use TRP and TRPL in similar amounts, whereas moths downregulate their TRP channel mRNAs. Because moths presumably have fewer TRP channels allowing in Ca^2+^, they also downregulate Na^+^/Ca^2+^ channels encoded by *Calx* and *Nckx30C*.

Phototransduction requires protein complexes to transduce and terminate the signal. One such complex is a target of G*α*q and is formed by INAD, TRP, PLC, and protein kinase C (Shieh et al. 1989; Chevesich et al. 1997; Tsunoda et al. 1997; Bähner et al. 2000; Montell 2005). *InaD* is required to localize and coordinate proteins in the phototransduction cascade to the microvillar membrane (Bähner et al. 2000). INAD and ninaC bind to each other, and individually bind calmodulin, which accelerates arrestin binding to rhodopsin to terminate phototransduction (Liu et al. 2008; Venkatachalam et al. 2010). Arrestin 1 and Arrestin 2 bind light-activated rhodopsin and discontinue cascade signaling in *D. melanogaster* (Dolph et al. 1993; Stavenga and Hardie 2011). Our data suggest that Arrestin 2 might be the major arrestin in butterfly phototransduction; it is more highly expressed than Arrestin 1 in moths as well (supplementary fig. S3*A*, Supplementary Material online). Phototransduction is also terminated by a protein with a suppressor of cytokine signaling box encoded by *stops*. The *stops* phenotype is associated with slow termination of phototransduction due to a decrease in *norpA* (PLC) (Wang et al. 2008). We find these genes to be upregulated in butterfly heads (Table 2), suggesting the actions of these complexes remain conserved. Lastly, Lepidoptera have a *ninaC2* gene, missing in *D. melanogaster*, which is upregulated in *H. melpomene* heads (supplementary fig. S5*G*, Supplementary Material online).

## Conclusions

Most studies of phototransduction in insects extrapolate from what is known in *D. melanogaster* to assign potential functions to genes based on sequence similarity. In our study, we used transcriptomics and phylogenetics to explore the conservation of phototransduction genes between *D. melanogaster* and Lepidoptera. We found that many orthologs of key *D. melanogaster* phototransduction genes were upregulated in *H. melpomene* heads relative to legs, antennae, and mouth parts. Our results suggest that many features of the *D. melanogaster* phototransduction cascade are conserved in lepidopteran vision. However, we found instances where lepidopteran paralogs are implicated in carrying out a visual role when an ortholog is lost. Differences in phototransduction between *D. melanogaster* and Lepidoptera occur in chromophore transport, chromophore regeneration, opsins, and DAG regulation. Although we found no conserved differences between moths and butterflies in gene gains and losses, quantifying gene expression in *M. sexta* and *H. melpomene* allowed us to detect differences in phototransduction between moths and butterflies. Notably, we found evidence that butterflies use both TRP and TRPL channels for phototransduction while moths downregulate *trp*, which is used for high light conditions (French et al. 2015). Along with decreased expression of *trp*, Na^+^/Ca^2+^ exchange channel mRNAs show decreased expression in nocturnal moths. We have thus completed the most extensive investigation of the evolution of the phototransduction cascade in Lepidoptera and have found that differences between Lepidoptera and *D. melanogaster* are due to gene gains and losses while differences between moths and butterflies are due to gene expression changes.

## Supplementary Material

Supplementary data are available at *Genome Biology and Evolution* online. 

## Supplementary Material

evz150_Supplementary_Data

## Notes

**Data deposition:** Phototransduction genes for *H. melpomene*, *M. sexta*, and *D. plexippus* were annotated and deposited in GenBank with accession numbers MK983015–MK983088, MK983089–MK983165, and MN037884–MN037955.
