# Supplementary material for: Evolution of Phototransduction Genes in Lepidoptera
Source: Genome Biol Evol. 2019 Jul 12;11(8):2107–24. doi: 10.1093/gbe/evz150 (PMC6698658; doi:10.1093/gbe/evz150)
Supplement: evz150_Supplementary_Data [file evz150_supplementary_data.zip › evz150_supplementary_data/Macias-Mun╠âoz_GBE_2019_Legends_Supplementary_Materials.docx]

**Legends supplementary materials**

**Table S1: Accession numbers for RNA-Seq data used.**

**Table S2: Number of phototransduction genes in eight insect genomes.**

**Table S3: *H. melpomene* BLAST results and annotations.**

**Table S4: *M. sexta* BLAST results and annotations.**

**Table S5: *D. plexippus* BLAST results and annotations.**

**Table S6: Phylogenetic tree models.**

**Table S7: Annotated DE contigs in head vs. antennae comparison.**

**Table S8: Annotated DE contigs in head vs. legs comparison.**

**Table S9: Annotated DE contigs in head vs. mouth parts comparison.**

**Table S10: Functional enrichment of differentially expressed genes.**

**Table S11: Annotated contigs commonly upregulated in heads.**

**Figure S1: Differential expression analysis.** (A) Multidimensional scaling (MDS) plot of RNA-seq libraries from *H. melpomene* antennae, heads, legs and mouth parts. The MDS plot shows that the heads group together and away from other tissues. (B) Heatmap of genes commonly upregulated in heads, numbers indicate log-fold change. Visual representation of expression confirms that the genes denoted as upregulated in heads by edgeR are highly expressed in heads and lowly expressed in other tissue types. (C) Level 2 biological process terms for genes commonly upregulated in heads using Blast2GO. (D) Multilevel pie chart summary of GO terms with node score information using Blast2GO. A node score is the number of sequences associated to with a particular GO term. One of the top terms includes G-protein coupled receptor signaling pathway (E) Enrichment results for genes commonly upregulated in heads and annotated using FlyBase and DAVID. The top three clusters show a function in light detection and phototransduction.

**Figure S2: Heatmaps for four tissue pair-wise comparisons.** (A) Heatmap for differentially expressed (DE) genes between head and antennae. (B) Heatmap for DE genes between head and legs. (C) Heatmap for DE genes between head and mouth parts. DE genes were found using a Bonferroni correction.

**Figure S3: Phylogeny and expression of phototransduction genes 1.** Phylogenetic trees were generated using amino acid sequences from eight insect genomes. Expression for all genes follows the convention boxed in red; orange bars represent expression in *M. sexta* heads, red bars expression *H. melpomene* heads, lilac expression in *H. melpomene* legs, green expression in *H. melpomene* antennae, and purple expression in *H. melpomene* mouth parts. (A) Arrestin (Arr) gene family. (B) Cacophony (cac) gene family. (C) Calx Na^+^/Ca^+^ exchange protein gene family. (D) Calmodulin (cam) gene family. Bars represent standard errors.

**Figure S4: Phylogeny and expression of phototransduction genes 2.** Phylogenetic trees were generated using amino acid sequences from eight insect genomes. Orange bars represent expression in *M. sexta* heads, red bars expression *H. melpomene* heads, lilac expression in *H. melpomene* legs, green expression in *H. melpomene* antennae, and purple expression in *H. melpomene* mouth parts. (A) CDP-diacylglycerol synthase (CdsA) gene family. (B) Dopa decarboxylase (Ddc) gene family. (C) Dual oxidase (Duox) gene family. (D) G protein alpha q (Galpha49B) gene family. (E) G protein beta (Gbeta76C) gene family. (F) G protein gamma (Ggamma30A) gene family. (G) G protein-coupled receptor kinase 1 (Gprk1) gene family. (H) G protein-coupled receptor kinase 2 (Gprk2) gene family. Bars represent standard errors.

**Figure S5: Phylogeny and expression of phototransduction genes 3.** Phylogenetic trees were generated using amino acid sequences from eight insect genomes. Orange bars represent expression in *M. sexta* heads, red bars expression *H. melpomene* heads, lilac expression in *H. melpomene* legs, green expression in *H. melpomene* antennae, and purple expression in *H. melpomene* mouth parts. (A) Inactivation no afterpotential D (InaD) gene family. (B) Nckx30C gene family. (C) Neither inactivation nor afterpotential A (ninaA) gene family. (D) NinaG gene family. (E) No receptor potential A gene (norpA) family. (F) Pyruvate dehydrogenase E1 beta (Pdhb) gene family. (G) Neither inactivation nor afterpotential C (ninaC) gene family. (H) RabX4 and Rab5 gene family. Bars represent standard errors.

**Figure S6: Phylogeny and expression of phototransduction genes 4.** Phylogenetic trees were generated using amino acid sequences from eight insect genomes. Orange bars represent expression in *M. sexta* heads, red bars expression *H. melpomene* heads, lilac expression in *H. melpomene* legs, green expression in *H. melpomene* antennae, and purple expression in *H. melpomene* mouth parts. (A) Phosphatidylinositol synthase (Pis) gene family. (B) Protein C kinase 53E (pkc53E) gene family. (C) Phospholipase D (Pld) gene family. (D) Retinal degeneration A (rdgA) gene family. (E) Retinal degeneration B (rdgB) gene family. (F) Retinal degeneration C (rdgC) gene family. (G) Vacuolar H+ ATPase 100kD subunit 1(Vha100) gene family. Bars represent standard errors.

**Figure S7: Phylogeny and expression of phototransduction genes 5.** Phylogenetic trees were generated using amino acid sequences from eight insect genomes. Orange bars represent expression in *M. sexta* heads, red bars expression *H. melpomene* heads, lilac expression in *H. melpomene* legs, green expression in *H. melpomene* antennae, and purple expression in *H. melpomene* mouth parts. (A) Innexin (inx/shakB/ogre) gene family. Bars represent standard errors.

**Figure S8: Immunohistochemistry of unclassified and long wavelength opsin.** Longitudinal sections of a butterfly retina. (A) Negative control for the unclassified opsin protein. For this experiment, the primary anti-UnRh was excluded only anti-LWRh and secondary antibodies were added to the sections to test for general background staining. (B) Diagonal section shows that UnRh is predominantly found in the upper 1/4^th^ of the butterfly ommatidia.
