# Supplementary material for: Evolution of Phototransduction Genes in Lepidoptera
Source: Genome Biol Evol. 2019 Jul 12;11(8):2107–24. doi: 10.1093/gbe/evz150 (PMC6698658; doi:10.1093/gbe/evz150)
Supplement: evz150_Supplementary_Data [file evz150_supplementary_data.zip › evz150_supplementary_data/Macias-Mun╠âoz_GBE_2019_Supplementary_Results.docx]

*Functional enrichment of genes differentially expressed between tissues*

We performed functional enrichment analyses for contigs DE between the 3 comparisons (head vs. legs, head vs. antennae, and head vs. mouth parts) to investigate the potential functions of these genes. We found that DE contigs for the three comparisons had some similar annotation clusters. Annotation terms that were similar across the three comparisons included detection of light stimulus, regulation of rhodopsin mediated signaling, and homeobox domain (Table S10). The first two annotation clusters included genes involved in phototransduction. The homeobox cluster included genes involved in antennal, leg and neuron development, as well as genes involved in compound eye development and morphogenesis such as *araucan*, *PvuII-PstI homology 13*, *ocelliless*, and *eyegone*. An annotation term unique to the head vs. antennae comparison was glucose-methanol-choline oxidoreductase which included the genes *glucose dehydrogenase* and *ninaG* among other yet unnamed genes (Table S10). An annotation term unique to the head vs. mouth parts comparison was ion channel activity and included genes involved in perception of touch, taste, and olfaction (Table S10). This cluster also included genes potentially involved in phototransduction such as *cacophony*, *NMDA receptor 1*, and *transient receptor potential-like* (*trpl*; Table S10).

*Loss of a Vha100 Duplicate in Non-Lepidopteran Insects*

A gene that was unique to Lepidoptera was *Vha100-like* (Figure S6G). In *Drosophila*, *Vacuolar H+ ATPase 100kD subunit* 1 (*Vha100-1*) encodes a V_0_ sector of a multisubunit complex, vesicular adenosine triphosphatase (v-ATPase) (Williamson et al. 2010). V-ATPase is involved in membrane fusion, acidification and synaptic vesicle exocytosis at photoreceptor presynaptic terminals (Williamson et al. 2010). Loss of *Vha100-1* results in a loss of transient response but overexpression results in photoreceptor cell death (Williamson et al. 2010). In our phylogenetic analysis of *Vha-100*, our results suggest that all insects except Lepidoptera have lost *Vha100-like* (Figure S6G). *Vha100-1*, *Vha100-like*, and *Vha100-2* are all expressed in *M. sexta* heads and in *H. melpomene* heads, antennae, legs, and mouth parts. None of the gene copies are upregulated in heads across all three comparisons but *Vha100-1* is upregulated in heads compared to leg and mouth. These results suggest that *Vha100-1* retains a visual function, but these genes may carry out additional functions in other tissues.

*Heliconius-specific innexin duplication*

Gap Junctions in vertebrates are composed of connexins and invertebrate gap junctions are composed of an unrelated protein family, innexins (Curtin et al. 2002; Bauer et al. 2005). In *Drosophila* it has been shown that the innexin family acts during development, where is expressed in its visual system and brain in different patterns and levels (Stebbings et al. 2002). *Ogre*, *shakB* and *inx2* are the genes of the best characterized innexins. *Ogre* (presynaptically) and *ShakB* (postsynaptically) are required for development of normal neural transmission (Curtin et al. 2002). *Inx2* participates in the regulation of morphogen-dependent organ size determination and is required broadly for embryonic epithelial morphogenesis (for this heteromerizes with Inx3) (Richard & Hoch, 2015). We looked for lepidopteran homologs of the *inx* family (from *inx1* to *inx8*) in transcriptomes and annotated genomes of Lepidoptera. After performing the alignment and phylogenetic tree of *inx* family we discovered a lepidopteran and a *Heliconius*-specific duplication in *inx9* (Figure S7). To confirm that inx9 was a *Heliconius*-specific duplication, we searched for the orthologs of this duplication in a closely related genus, *Melitaea cinxia* and were not able to find an ortholog in *M. cinxia.* In order to understand the role of the *Heliconius* duplications, their expression levels were analyzed together with the other *inx* family members (Figure S7). Thus, we found that *inx9* and *inx9-like* had expression in heads but the expression was not higher in heads relative to other tissues.

**References:**

Bauer R et al. 2005. Intercellular communication: The *Drosophila* innexin multiprotein family of gap junction proteins. Chem. Biol. 12:515–526. doi: 10.1016/j.chembiol.2005.02.013.

Curtin KD, Zhang Z, Wyman RJ. 2002. Gap junction proteins are not interchangeable in development of neural function in the *Drosophila* visual system. J. Cell Sci. 115:3379–3388.

Stebbings LA et al. 2002. Gap junctions in *Drosophila*: Developmental expression of the entire innexin gene family. Mech. Dev. 113:197–205. doi: 10.1016/S0925-4773(02)00025-4.

Williamson WR, Wang D, Haberman AS, Hiesinger PR. 2010. A dual function of V0-ATPase a1 provides an endolysosomal degradation mechanism in *Drosophila melanogaster* photoreceptors. J. Cell Biol. 189:885–899. doi: 10.1083/jcb.201003062.
