# Supplementary material for: Evolution of Phototransduction Genes in Lepidoptera
Source: Genome Biol Evol. 2019 Jul 12;11(8):2107–24. doi: 10.1093/gbe/evz150 (PMC6698658; doi:10.1093/gbe/evz150)
Supplement: evz150_Supplementary_Data [file evz150_supplementary_data.zip › evz150_supplementary_data/Macias-Mun╠âoz_GBE_2019_FigS6.pdf]

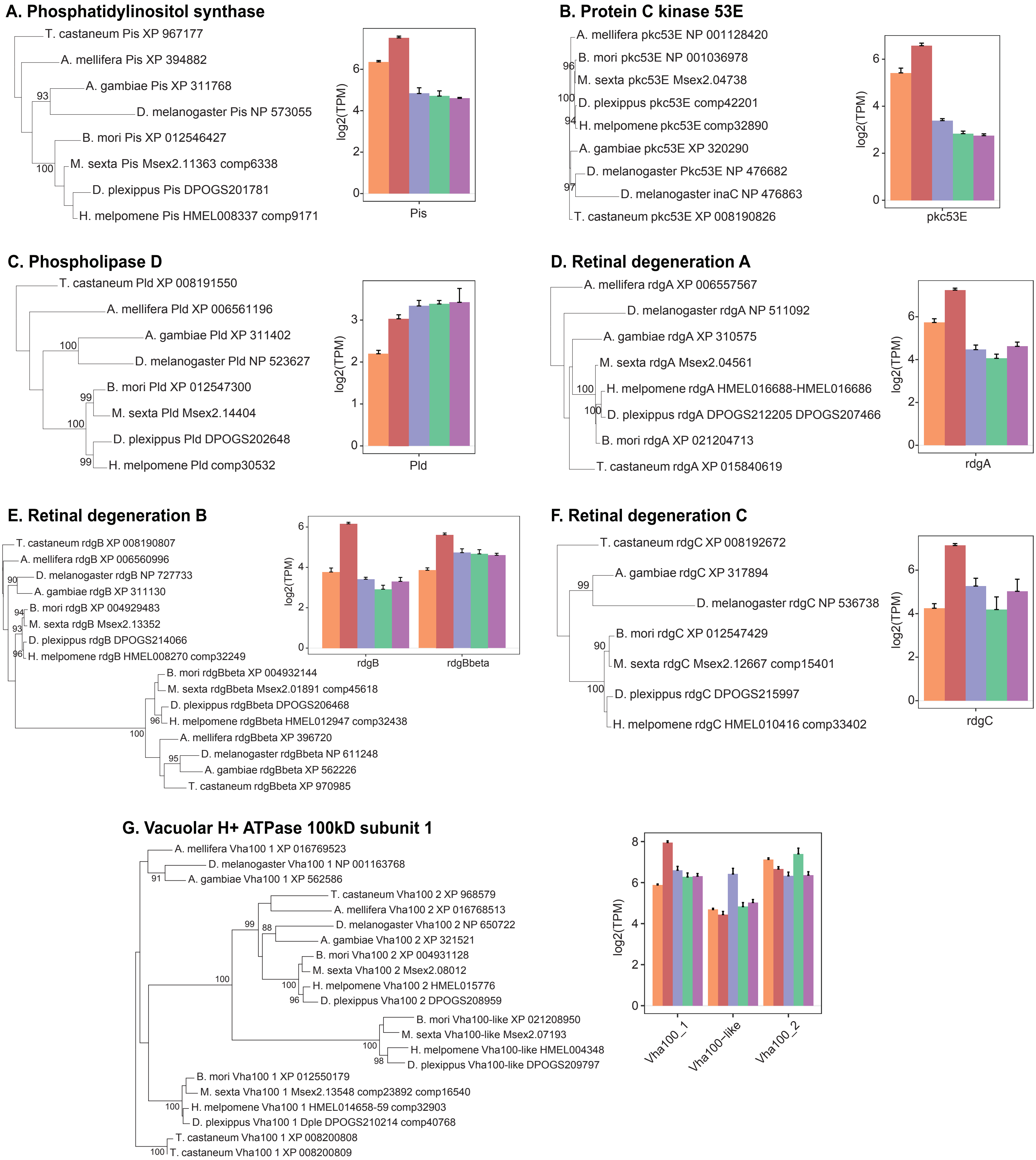
A. Phosphatidylinositol synthase
T. castaneum Pis XP 967177
A. mellifera Pis XP 394882
A. gambiae Pis XP 311768
D. melanogaster Pis NP 573055
B. mori Pis XP 012546427
M. sexta Pis Msex2.11363 comp6338
D. plexippus Pis DPOGS201781
H. melpomene Pis HMEL008337 comp9171
93
100
log2(TPM)
Pis
B. Protein C kinase 53E
A. mellifera pkc53E NP 001128420
B. mori pkc53E NP 001036978
M. sexta pkc53E Msex2.04738
D. plexippus pkc53E comp42201
H. melpomene pkc53E comp32890
A. gambiae pkc53E XP 320290
D. melanogaster Pkc53E NP 476682
D. melanogaster inaC NP 476863
T. castaneum pkc53E XP 008190826
96
100
94
97
log2(TPM)
pkc53E
C. Phospholipase D
T. castaneum Pld XP 008191550
A. mellifera Pld XP 006561196
A. gambiae Pld XP 311402
D. melanogaster Pld NP 523627
B. mori Pld XP 012547300
M. sexta Pld Msex2.14404
D. plexippus Pld DPOGS202648
H. melpomene Pld comp30532
100
99
100
99
log2(TPM)
Pld
D. Retinal degeneration A
A. mellifera rdgA XP 006557567
D. melanogaster rdgA NP 511092
A. gambiae rdgA XP 310575
M. sexta rdgA Msex2.04561
H. melpomene rdgA HMEL016688-HMEL016686
D. plexippus rdgA DPOGS212205 DPOGS207466
B. mori rdgA XP 021204713
T. castaneum rdgA XP 015840619
100
100
log2(TPM)
rdgA
E. Retinal degeneration B
T. castaneum rdgB XP 008190807
A. mellifera rdgB XP 006560996
D. melanogaster rdgB NP 727733
A. gambiae rdgB XP 311130
B. mori rdgB XP 004929483
M. sexta rdgB Msex2.13352
D. plexippus rdgB DPOGS214066
H. melpomene rdgB HMEL008270 comp32249
B. mori rdgBbeta XP 004932144
M. sexta rdgBbeta Msex2.01891 comp45618
D. plexippus rdgBbeta DPOGS206468
H. melpomene rdgBbeta HMEL012947 comp32438
A. mellifera rdgBbeta XP 396720
D. melanogaster rdgBbeta NP 611248
A. gambiae rdgBbeta XP 562226
T. castaneum rdgBbeta XP 970985
90
94
93
96
96
100
95
log2(TPM)
rdgB
rdgBbeta
F. Retinal degeneration C
T. castaneum rdgC XP 008192672
A. gambiae rdgC XP 317894
D. melanogaster rdgC NP 536738
B. mori rdgC XP 012547429
M. sexta rdgC Msex2.12667 comp15401
D. plexippus rdgC DPOGS215997
H. melpomene rdgC HMEL010416 comp33402
99
90
100
log2(TPM)
rdgC
G. Vacuolar H+ ATPase 100kD subunit 1
A. mellifera Vha100 1 XP 016769523
D. melanogaster Vha100 1 NP 001163768
A. gambiae Vha100 1 XP 562586
T. castaneum Vha100 2 XP 968579
A. mellifera Vha100 2 XP 016768513
D. melanogaster Vha100 2 NP 650722
A. gambiae Vha100 2 XP 321521
B. mori Vha100 2 XP 004931128
M. sexta Vha100 2 Msex2.08012
H. melpomene Vha100 2 HMEL015776
D. plexippus Vha100 2 DPOGS208959
B. mori Vha100-like XP 021208950
M. sexta Vha100-like Msex2.07193
H. melpomene Vha100-like HMEL004348
D. plexippus Vha100-like DPOGS209797
B. mori Vha100 1 XP 012550179
M. sexta Vha100 1 Msex2.13548 comp23892 comp16540
H. melpomene Vha100 1 HMEL014658-59 comp32903
D. plexippus Vha100 1 Dple DPOGS210214 comp40768
T. castaneum Vha100 1 XP 008200808
T. castaneum Vha100 1 XP 008200809
91
99
88
100
100
96
100
98
100
100
log2(TPM)
Vha100_1
Vha100–like
Vha100_2
