# Supplementary material for: Evolution of Phototransduction Genes in Lepidoptera
Source: Genome Biol Evol. 2019 Jul 12;11(8):2107–24. doi: 10.1093/gbe/evz150 (PMC6698658; doi:10.1093/gbe/evz150)
Supplement: evz150_Supplementary_Data [file evz150_supplementary_data.zip › evz150_supplementary_data/Macias-Mun╠âoz_GBE_2019_FigS5.pdf]

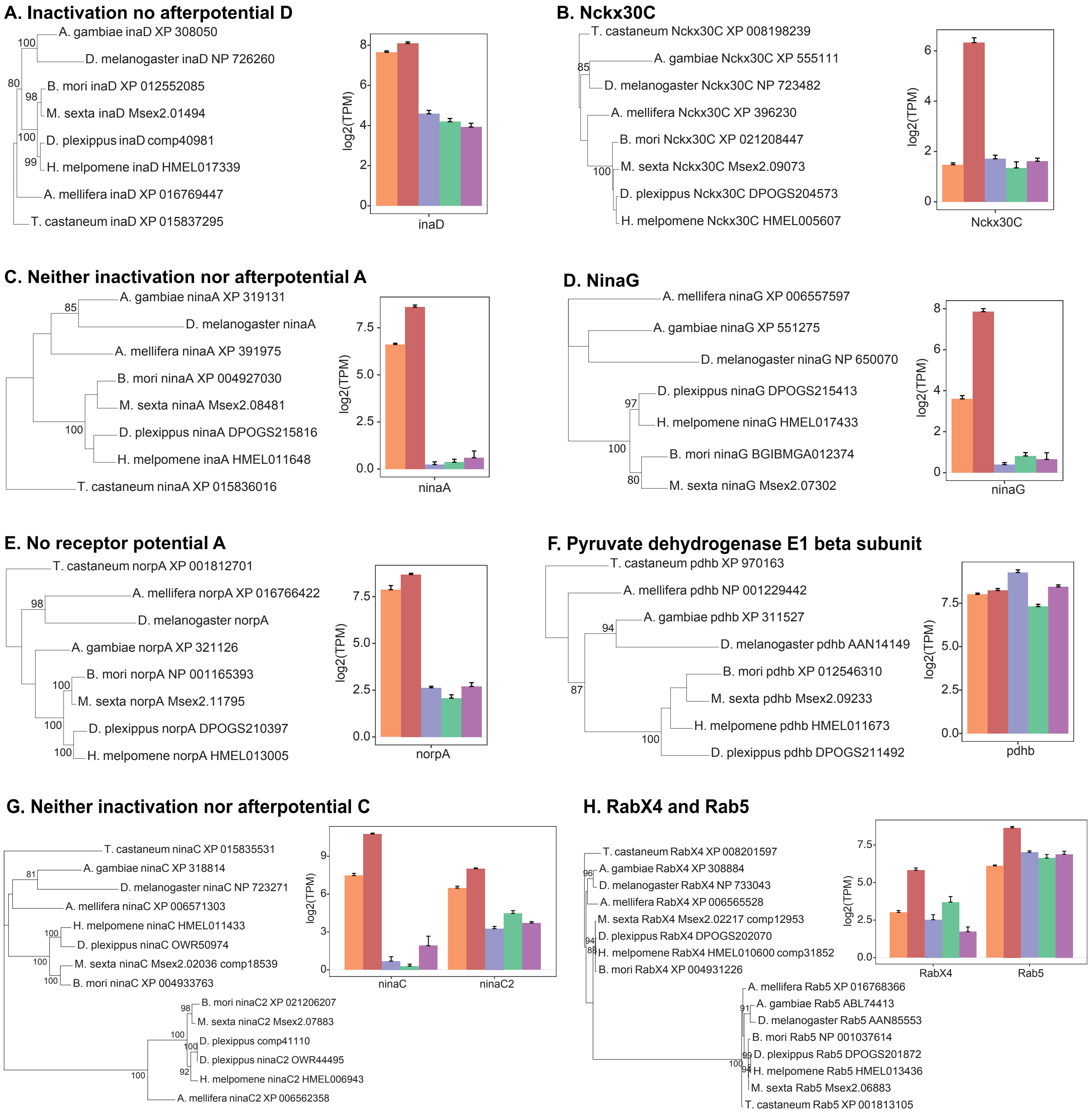

A. Inactivation no afterpotential D
A. gambiae inaD XP 308050
D. melanogaster inaD NP 726260
B. mori inaD XP 012552085
M. sexta inaD Msex2.01494
D. plexippus inaD comp40981
H. melpomene inaD HMEL017339
A. mellifera inaD XP 016769447
T. castaneum inaD XP 015837295
100
80
98
100
99
log2(TPM)
inaD
B. Nckx30C
T. castaneum Nckx30C XP 008198239
A. gambiae Nckx30C XP 555111
D. melanogaster Nckx30C NP 723482
A. mellifera Nckx30C XP 396230
B. mori Nckx30C XP 021208447
M. sexta Nckx30C Msex2.09073
D. plexippus Nckx30C DPOGS204573
H. melpomene Nckx30C HMEL005607
85
100
log2(TPM)
Nckx30C
C. Neither inactivation nor afterpotential A
A. gambiae ninaA XP 319131
D. melanogaster ninaA
A. mellifera ninaA XP 391975
B. mori ninaA XP 004927030
M. sexta ninaA Msex2.08481
D. plexippus ninaA DPOGS215816
H. melpomene inaA HMEL011648
T. castaneum ninaA XP 015836016
85
100
log2(TPM)
ninaA
D. NinaG
A. mellifera ninaG XP 006557597
A. gambiae ninaG XP 551275
D. melanogaster ninaG NP 650070
D. plexippus ninaG DPOGS215413
H. melpomene ninaG HMEL017433
B. mori ninaG BGIBMGA012374
M. sexta ninaG Msex2.07302
97
100
80
log2(TPM)
ninaG
E. No receptor potential A
T. castaneum norpA XP 001812701
A. mellifera norpA XP 016766422
D. melanogaster norpA
A. gambiae norpA XP 321126
B. mori norpA NP 001165393
M. sexta norpA Msex2.11795
D. plexippus norpA DPOGS210397
H. melpomene norpA HMEL013005
98
100
100
100
log2(TPM)
norpA
F. Pyruvate dehydrogenase E1 beta subunit
T. castaneum pdhb XP 970163
A. mellifera pdhb NP 001229442
A. gambiae pdhb XP 311527
D. melanogaster pdhb AAN14149
B. mori pdhb XP 012546310
M. sexta pdhb Msex2.09233
H. melpomene pdhb HMEL011673
D. plexippus pdhb DPOGS211492
94
87
100
log2(TPM)
pdhb
G. Neither inactivation nor afterpotential C
T. castaneum ninaC XP 015835531
A. gambiae ninaC XP 318814
D. melanogaster ninaC NP 723271
A. mellifera ninaC XP 006571303
H. melpomene ninaC HMEL011433
D. plexippus ninaC OWR50974
M. sexta ninaC Msex2.02036 comp18539
B. mori ninaC XP 004933763
B. mori ninaC2 XP 021206207
M. sexta ninaC2 Msex2.07883
D. plexippus comp41110
D. plexippus ninaC2 OWR44495
H. melpomene ninaC2 HMEL006943
A. mellifera ninaC2 XP 006562358
81
100
100
100
98
100
100
92
100
log2(TPM)
ninaC
ninaC2
H. RabX4 and Rab5
T. castaneum RabX4 XP 008201597
A. gambiae RabX4 XP 308884
D. melanogaster RabX4 NP 733043
A. mellifera RabX4 XP 006565528
M. sexta RabX4 Msex2.02217 comp12953
D. plexippus RabX4 DPOGS202070
H. melpomene RabX4 HMEL010600 comp31852
B. mori RabX4 XP 004931226
A. mellifera Rab5 XP 016768366
A. gambiae Rab5 ABL74413
D. melanogaster Rab5 AAN85553
B. mori Rab5 NP 001037614
D. plexippus Rab5 DPOGS201872
H. melpomene Rab5 HMEL013436
M. sexta Rab5 Msex2.06883
T. castaneum Rab5 XP 001813105
96
94
88
91
99
100
94
log2(TPM)
RabX4
Rab5
