# Supplementary material for: Evolution of Phototransduction Genes in Lepidoptera
Source: Genome Biol Evol. 2019 Jul 12;11(8):2107–24. doi: 10.1093/gbe/evz150 (PMC6698658; doi:10.1093/gbe/evz150)
Supplement: evz150_Supplementary_Data [file evz150_supplementary_data.zip › evz150_supplementary_data/Macias-Mun╠âoz_GBE_2019_FigS4.pdf]

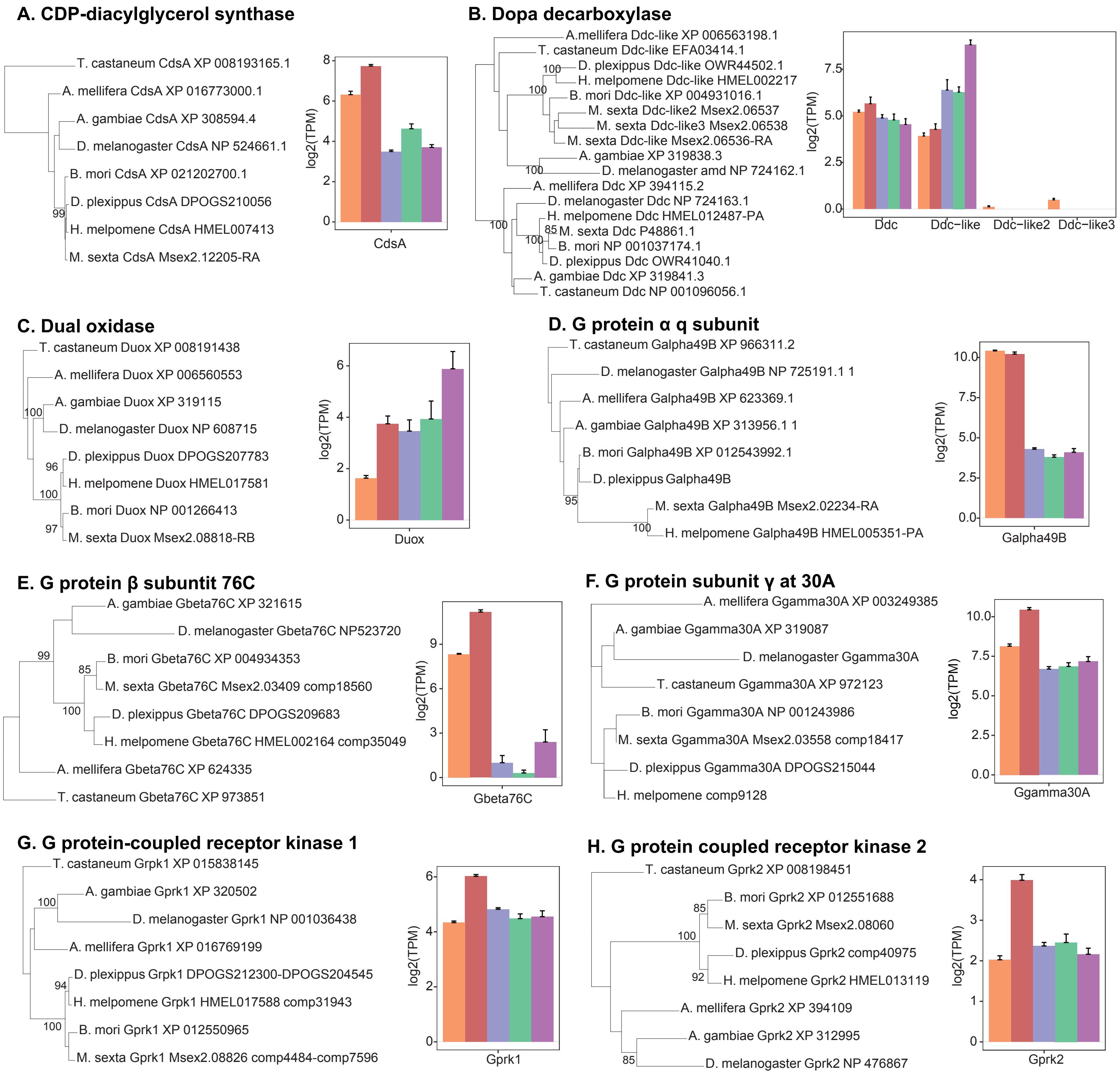
A. CDP-diacylglycerol synthase
T. castaneum CdsA XP 008193165.1
A. mellifera CdsA XP 016773000.1
A. gambiae CdsA XP 308594.4
D. melanogaster CdsA NP 524661.1
B. mori CdsA XP 021202700.1
D. plexippus CdsA DPOGS210056
99
H. melpomene CdsA HMEL007413
M. sexta CdsA Msex2.12205-RA
log2(TPM)
CdsA
B. Dopa decarboxylase
A.mellifera Ddc-like XP 006563198.1
T. castaneum Ddc-like EFA03414.1
D. plexippus Ddc-like OWR44502.1
H. melpomene Ddc-like HMEL002217
B. mori Ddc-like XP 004931016.1
M. sexta Ddc-like2 Msex2.06537
M. sexta Ddc-like3 Msex2.06538
M. sexta Ddc-like Msex2.06536-RA
A. gambiae XP 319838.3
D. melanogaster amd NP 724162.1
A. mellifera Ddc XP 394115.2
D. melanogaster Ddc NP 724163.1
H. melpomene Ddc HMEL012487-PA
M. sexta Ddc P48861.1
B. mori NP 001037174.1
D. plexippus Ddc OWR41040.1
A. gambiae Ddc XP 319841.3
T. castaneum Ddc NP 001096056.1
log2(TPM)
Ddc
Ddc−like
Ddc−like2
Ddc−like3
C. Dual oxidase
T. castaneum Duox XP 008191438
A. mellifera Duox XP 006560553
A. gambiae Duox XP 319115
D. melanogaster Duox NP 608715
D. plexippus Duox DPOGS207783
H. melpomene Duox HMEL017581
B. mori Duox NP 001266413
M. sexta Duox Msex2.08818-RB
log2(TPM)
Duox
D. G protein α q subunit
T. castaneum Galpha49B XP 966311.2
D. melanogaster Galpha49B NP 725191.1 1
A. mellifera Galpha49B XP 623369.1
A. gambiae Galpha49B XP 313956.1 1
B. mori Galpha49B XP 012543992.1
D. plexippus Galpha49B
M. sexta Galpha49B Msex2.02234-RA
H. melpomene Galpha49B HMEL005351-PA
log2(TPM)
Galpha49B
E. G protein β subuntit 76C
A. gambiae Gbeta76C XP 321615
D. melanogaster Gbeta76C NP523720
B. mori Gbeta76C XP 004934353
M. sexta Gbeta76C Msex2.03409 comp18560
D. plexippus Gbeta76C DPOGS209683
H. melpomene Gbeta76C HMEL002164 comp35049
A. mellifera Gbeta76C XP 624335
T. castaneum Gbeta76C XP 973851
log2(TPM)
Gbeta76C
F. G protein subunit γ at 30A
A. mellifera Ggamma30A XP 003249385
A. gambiae Ggamma30A XP 319087
D. melanogaster Ggamma30A
T. castaneum Ggamma30A XP 972123
B. mori Ggamma30A NP 001243986
M. sexta Ggamma30A Msex2.03558 comp18417
D. plexippus Ggamma30A DPOGS215044
H. melpomene comp9128
log2(TPM)
Ggamma30A
G. G protein-coupled receptor kinase 1
T. castaneum Grpk1 XP 015838145
A. gambiae Grpk1 XP 320502
D. melanogaster Gprk1 NP 001036438
A. mellifera Gprk1 XP 016769199
D. plexippus Grpk1 DPOGS212300-DPOGS204545
H. melpomene Grpk1 HMEL017588 comp31943
B. mori Gprk1 XP 012550965
M. sexta Gprk1 Msex2.08826 comp4484-comp7596
log2(TPM)
Gprk1
H. G protein coupled receptor kinase 2
T. castaneum Gprk2 XP 008198451
B. mori Gprk2 XP 012551688
M. sexta Gprk2 Msex2.08060
D. plexippus Gprk2 comp40975
H. melpomene Gprk2 HMEL013119
A. mellifera Gprk2 XP 394109
A. gambiae Gprk2 XP 312995
D. melanogaster Gprk2 NP 476867
log2(TPM)
Gprk2
