# Supplementary figures and images for: Evolution of Phototransduction Genes in Lepidoptera
Source: Genome Biol Evol. 2019 Jul 12;11(8):2107–24. doi: 10.1093/gbe/evz150 (PMC6698658; doi:10.1093/gbe/evz150)

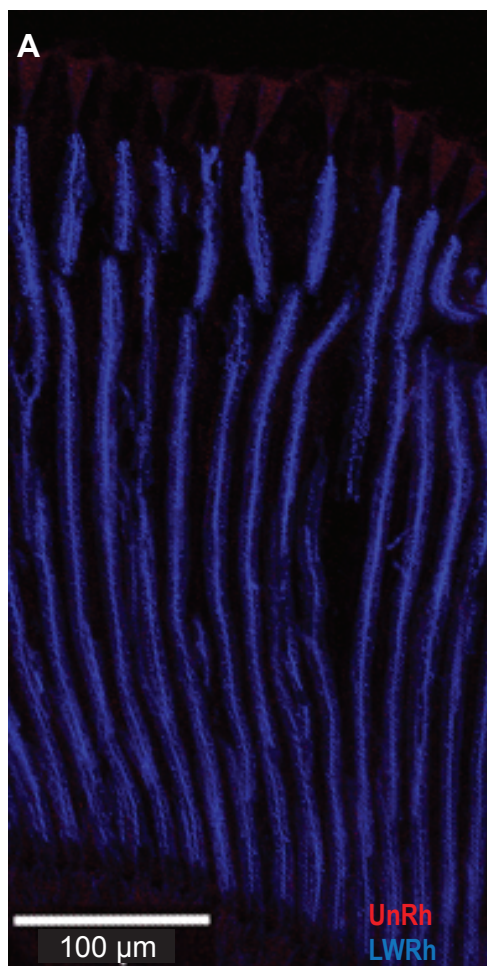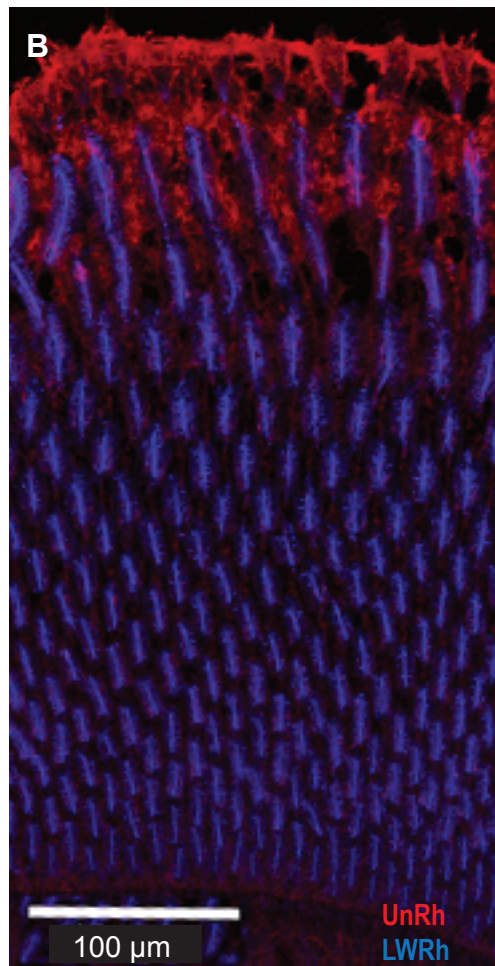

Supplement: evz150_Supplementary_Data [file evz150_supplementary_data.zip › evz150_supplementary_data/Macias-Mun╠âoz_GBE_2019_FigS8.pdf]

## A. Innexin

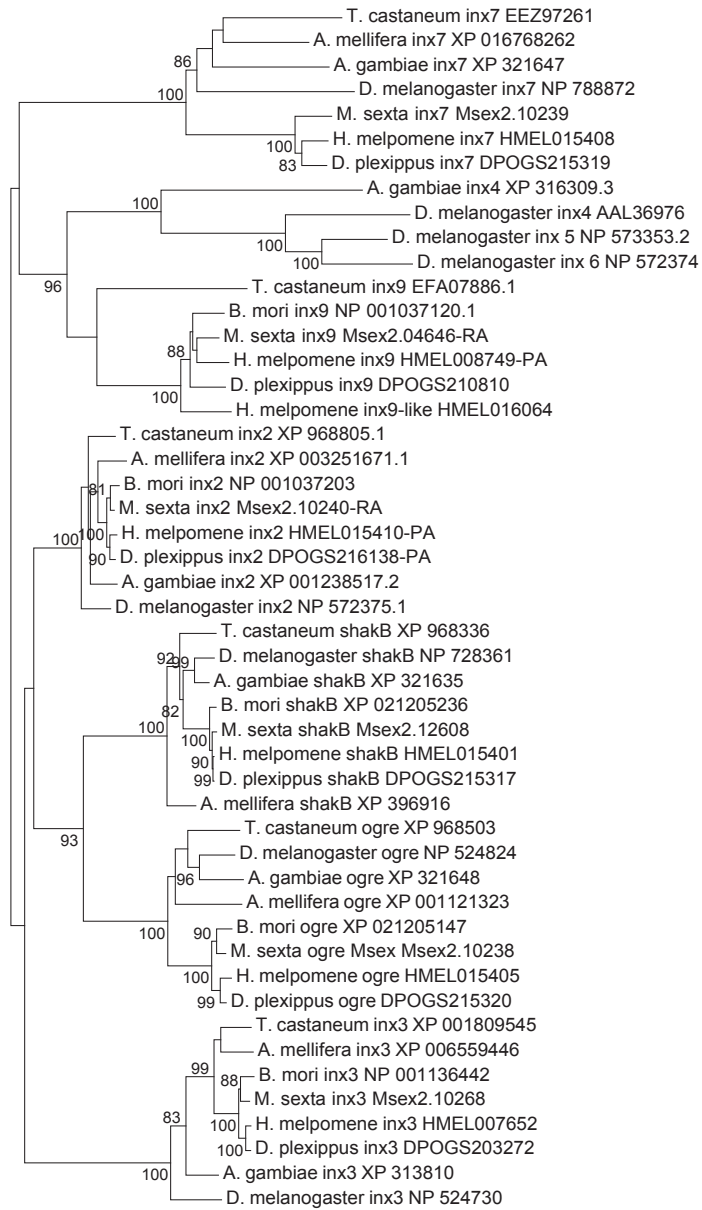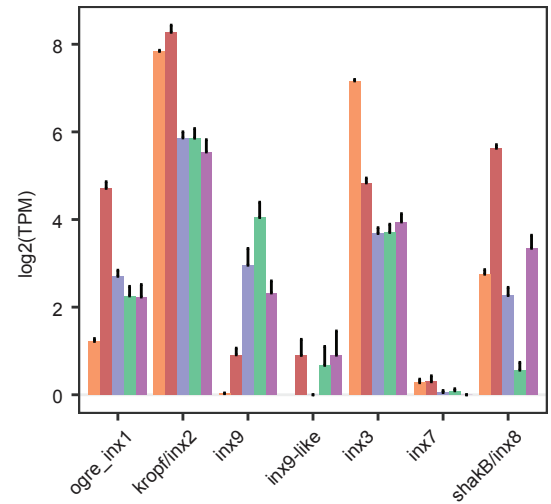

Supplement: evz150_Supplementary_Data [file evz150_supplementary_data.zip › evz150_supplementary_data/Macias-Mun╠âoz_GBE_2019_FigS7.pdf]

A. Arrestin

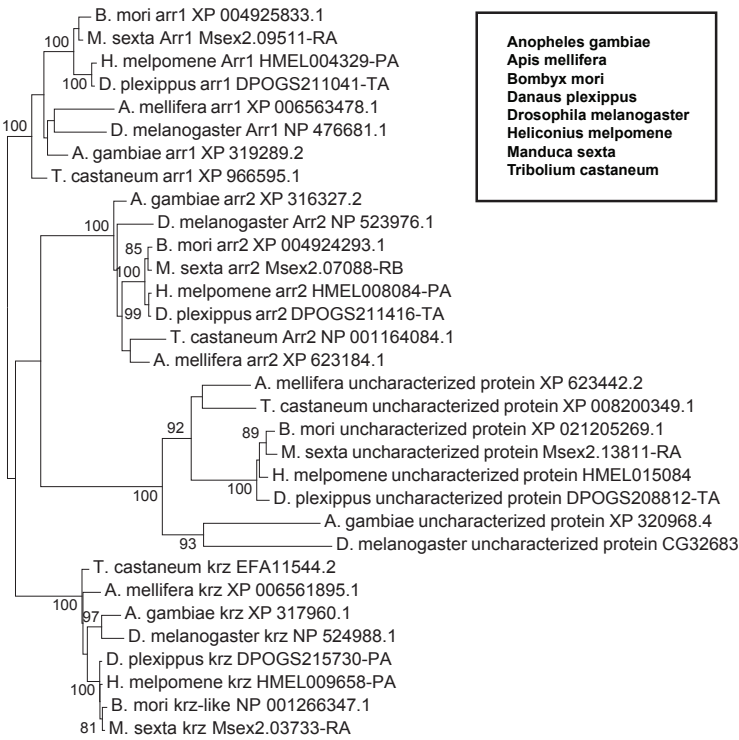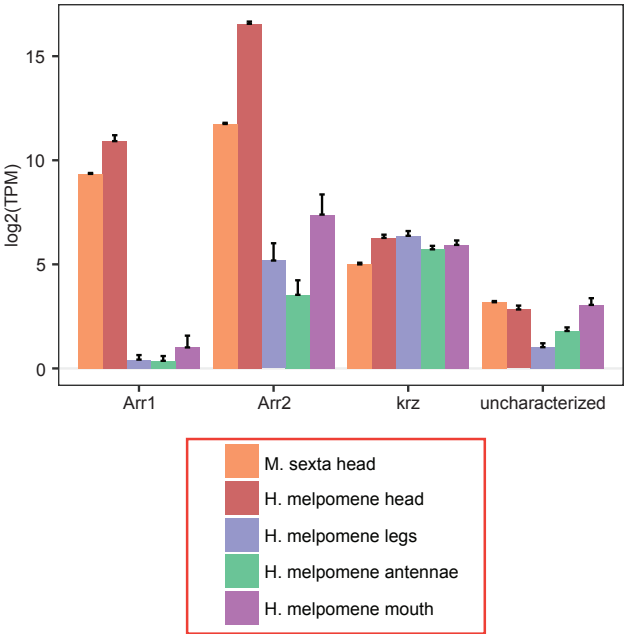

B. Cacophony

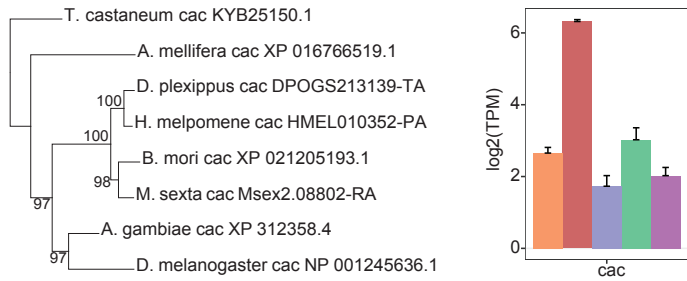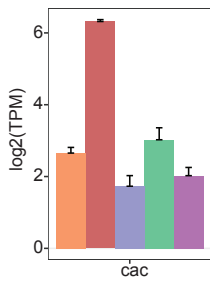

C. Calx Na/Ca-exchange protein

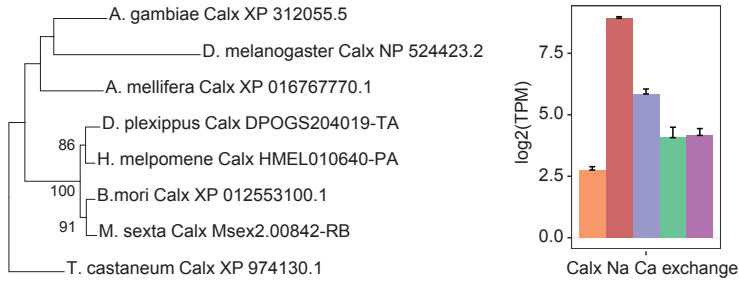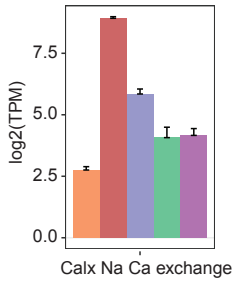

D. Calmodulin

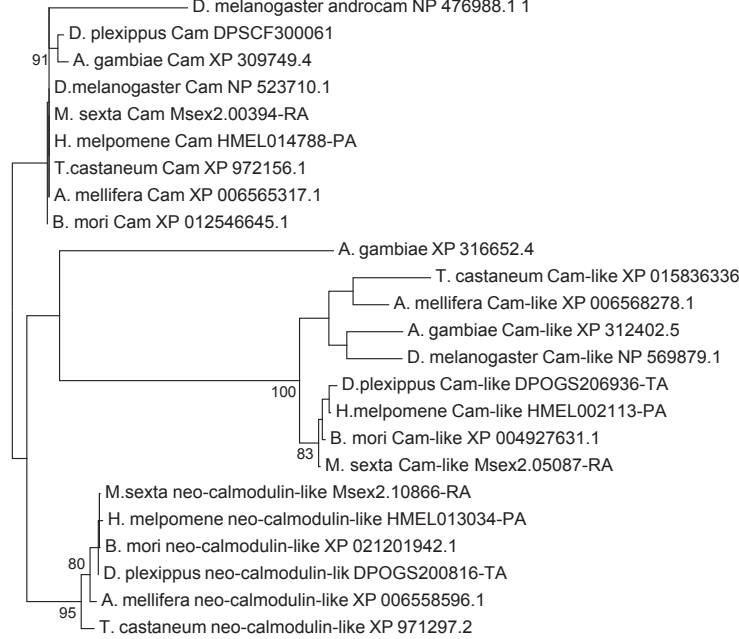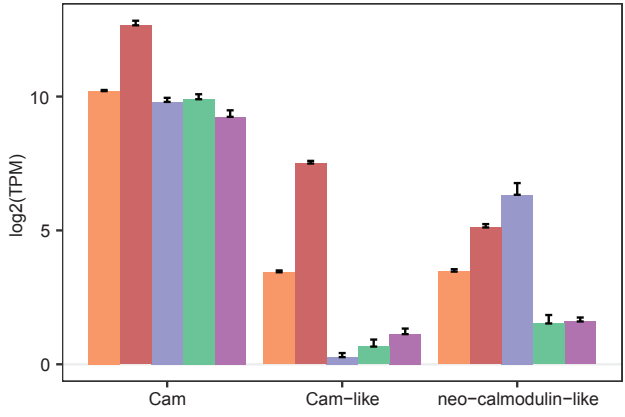

Supplement: evz150_Supplementary_Data [file evz150_supplementary_data.zip › evz150_supplementary_data/Macias-Mun╠âoz_GBE_2019_FigS3.pdf]

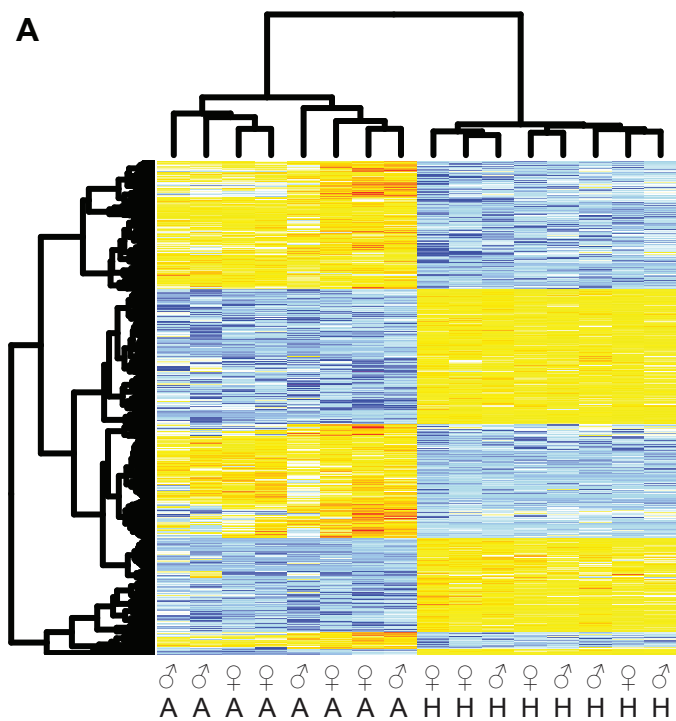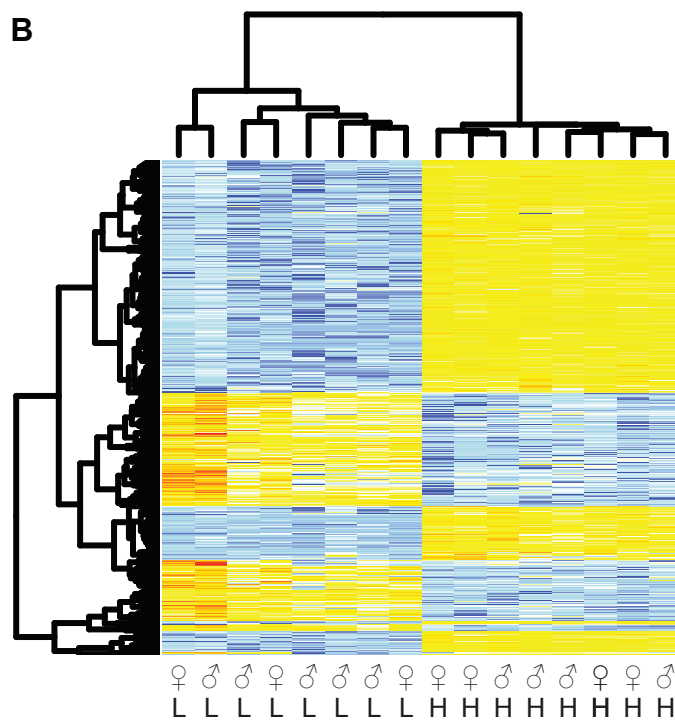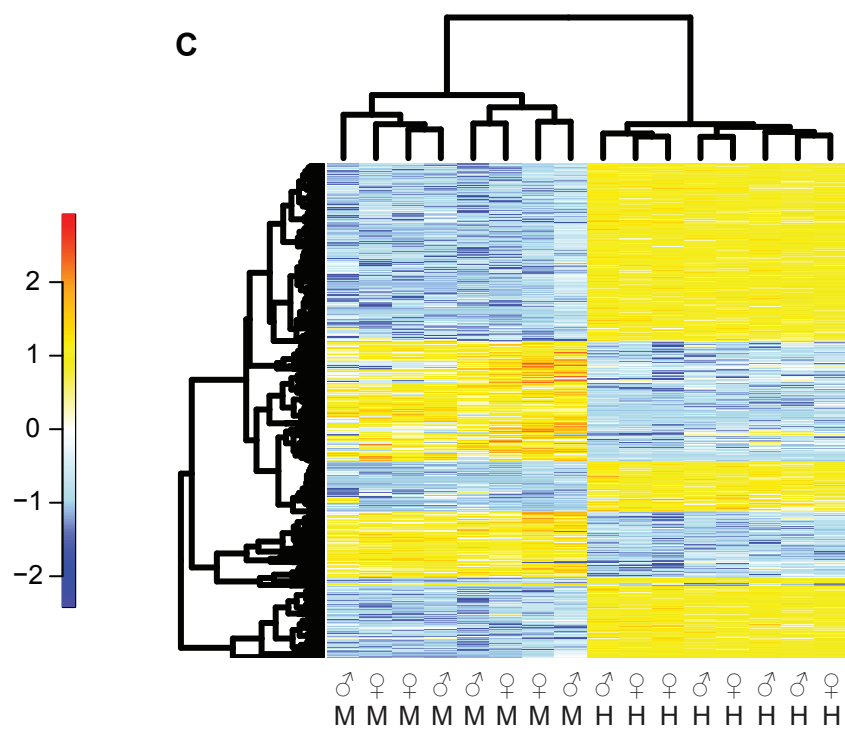

Supplement: evz150_Supplementary_Data [file evz150_supplementary_data.zip › evz150_supplementary_data/Macias-Mun╠âoz_GBE_2019_FigS2.pdf]
