# Supplementary material for: Evolution of Phototransduction Genes in Lepidoptera
Source: Genome Biol Evol. 2019 Jul 12;11(8):2107–24. doi: 10.1093/gbe/evz150 (PMC6698658; doi:10.1093/gbe/evz150)
Supplement: evz150_Supplementary_Data [file Macias-Mun╠âoz_GBE_2019_FigS1.pdf › evz150_supplementary_data/Macias-Mun╠âoz_GBE_2019_FigS1.pdf]

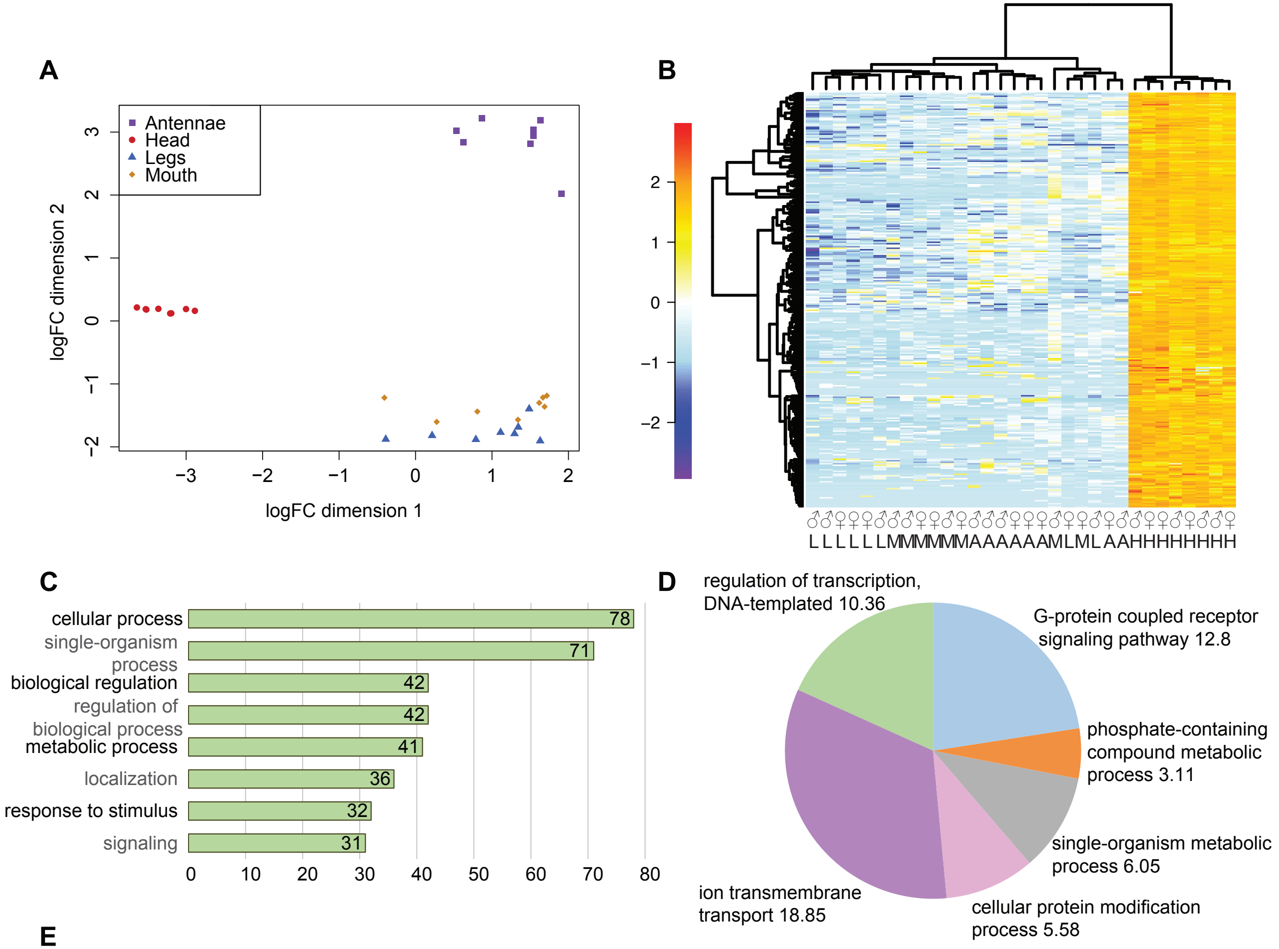

E

Annotation Cluster	Enrichment Score	Term	Count	Genes	PValue	Bonferroni
1	9.757	detection of light stimulus	12	NINAC, INAD, CALX, ARR2, ARR1, RH3, RH5 STOPS, TRPL, NORPA, GBETA76C, NINAA	1.85E-12	1.06E-09
2	8.134	regulation of rhodopsin mediated signaling pathway	7	NINAC, INAD, ARR2, ARR1, STOPS, NORPA, GBETA76C	2.44E-09	1.40E-06
3	8.077	detection of light stimulus involved in visual perception	8	NINAC, INAD, ARR2, ARR1, STOPS, TRPL, NORPA, GBETA76C	8.40E-10	4.81E-07
4	6.062	ligand-gated ion channel activity	11	RDL, CG8916, NMDAR1, LCCH3, HISCL1, ORT, TRPL, CG42260, CG3822, CG11155, NMDAR2	2.47E-09	5.83E-07
5	3.739	Gamma-aminobutyric acid A receptor	5	RDL, CG8916, LCCH3, HISCL1, ORT	5.94E-06	0.001
6	3.085	synaptic transmission	9	IPP, SYT1, NMDAR1, CPX, ORT, CG1909, GAD1, VGLUT, SIF	5.97E-04	0.290
7	2.715	NMDA receptor	4	NMDAR1, CG11155, NMDAR2, CG3822	3.23E-04	0.069
8	2.652	chloride channel complex	4	RDL, LCCH3, HISCL1, ORT	4.90E-04	0.049
9	2.307	Homeobox	6	CG11294, BSH, HMX, PPH13, SCRO, OC	0.002	0.287
10	1.288	rhabdomere development	4	PPH13, OC, PROM, CHP	0.001	0.532
11	1.144	C2 membrane targeting protein	3	SYT1, SL, NORPA	0.046	1.000
